# Visualizing cellular imaging data using PhenoPlot

**DOI:** 10.1038/ncomms6825

**Published:** 2015-01-08

**Authors:** Heba Z. Sailem, Julia E. Sero, Chris Bakal

**Affiliations:** 1 Dynamical Cell Systems, Division of Cancer Biology, Institute of Cancer Research, 237 Fulham Road, London SW3 6JB, UK

## Abstract

Visualization is essential for data interpretation, hypothesis formulation and communication of results. However, there is a paucity of visualization methods for image-derived data sets generated by high-content analysis in which complex cellular phenotypes are described as high-dimensional vectors of features. Here we present a visualization tool, PhenoPlot, which represents quantitative high-content imaging data as easily interpretable glyphs, and we illustrate how PhenoPlot can be used to improve the exploration and interpretation of complex breast cancer cell phenotypes.

## Abstract (web summary)

Cellular imaging studies can generate large volumes of complex phenotypic data; however, presenting this information in a form that quickly conveys trends in the data set remains a challenge. Sailem *et al.* present a tool which translates such data into easily interpretable cell-like glyphs.

## Graphical abstract

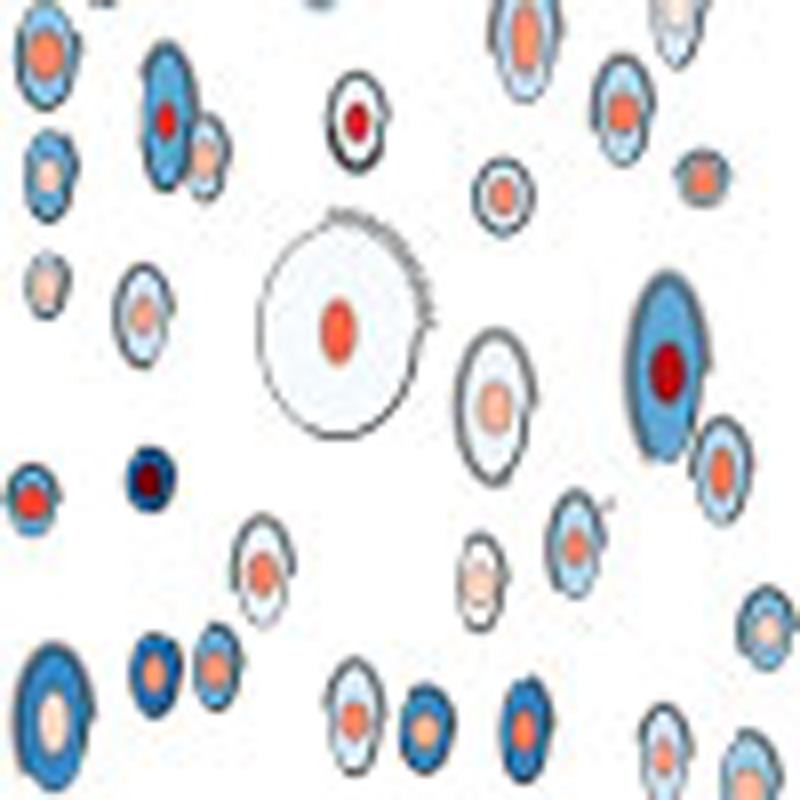

Using microscopy and computer vision methods, researchers can now quantify cellular and subcellular phenotypes, signalling states, and the spatial organization of single cells^1^,^2^. Detecting and describing the similarities and differences between cellular phenotypes becomes increasingly difficult as the number of cell images increases, even when the images and the quantification of these images are available. For example, while the manual examination of raw images can often detect subtle differences in phenotypes, human beings are prone to bias and such differences may or may not exist numerically. This means that a disparity may exist between what the observer believes to be the phenotype, and the quantitative phenotype itself. Conversely, an experimentalist may not be able to see some of the phenotypic differences that are detected by computational analysis in raw images, as humans cannot easily discern aspects such as pixel intensities, ‘texture’ (distribution of pixel intensities) and subtle changes in label localization^3^. Moreover, many images may be acquired across different channels, which increases the number of dimensions the analyst needs to work with. Finally, it is difficult for observers to appreciate how different features, such as area, shape and the intensity of different labels are quantitatively related to each other. Thus, the success of any image-based study relies heavily on the ability of the experimentalist to relate images with numerical data.

Visualization can greatly facilitate data analysis and interpretation, which are still major bottlenecks in gaining biologically meaningful knowledge from imaging data. Coordinate-based graphs and heatmaps^4^ are the most frequently used methods for representing imaging measurements, but they have a number of drawbacks. Coordinate-based graphs such as bar charts and scatter plots are restricted to three dimensions, while parallel coordinates can represent many dimensions but may suffer from occlusion between data points^5^. On the other hand, heatmaps use coloured objects (typically boxes) to represent many dimensions^4^, but it can be difficult for humans to discern the extent to which different hues reflect differences in phenotypes^6^. Critically in the context of image-based data sets, neither coordinate-based graphs nor heatmaps are intuitive representations of cellular phenotypes, as they do not use pictorial representations of individual features. It may therefore be difficult for experimentalists to understand what any given cell or population looks like, or to relate numbers to images, using heatmaps or scatter plots.

Glyph-based methods use a collection of visual elements such as size, colour, texture and/or orientation to depict multidimensional data^7^. For example, star glyphs use radial bars with length proportional to variable values^8^. Another example is the facial glyphs proposed by Chernoff *et al.*^8^,^9^ to represent around 20 variables. Chernoff faces exploit humans’ ability to detect differences between faces and allow quick identification of which samples are different from one another. However, facial glyphs are rarely used to represent cellular data because they imply emotional expressions that might not be relevant to the represented data^8^,^10^. Like other visualization methods, the available glyph-based methods are not intuitive representations of cellular phenotypes and do not address the question of how samples differ in terms of their visual phenotypes.

We design and develop PhenoPlot, a glyph-based approach, to represent multidimensional cellular measurements in an intuitive manner. PhenoPlot is a free and open source Matlab toolbox that comes with a graphical user interface (GUI). Currently, PhenoPlot allows the visualization of up to 21 variables. We illustrate the utility of PhenoPlot in profiling the morphology of breast cancer cell lines and show how PhenoPlot can be a useful tool in understanding and interpreting multidimensional cellular imaging data.

## Results

### PhenoPlot design

**Figure 1:**
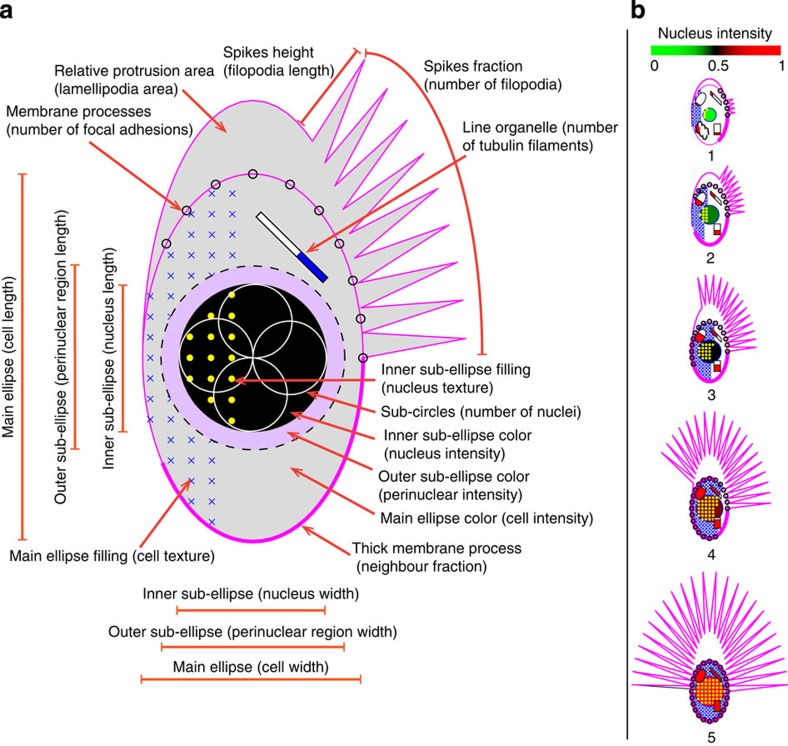
PhenoPlot design. (**a**) Illustration of the main visualization elements in PhenoPlot. Each element can represent a feature quantified from raw image data. Examples of the features that these elements can represent are shown in parentheses. (**b**) PhenoPlots of 15 variables. The PhenoPlot elements used here are main ellipse (length and width), main ellipse filling, inner sub-ellipse (length and width), inner sub-ellipse filling, inner sub-ellipse colour, relative protrusion area, spikes (fraction and height), membrane process, line organelle, ellipse organelle and rectangle organelle as detailed in Supplementary Table 2.

PhenoPlot employs many visual elements, such as differently sized, coloured and structured objects, to represent multiple dimensions independently of XY coordinates. Supplementary Table 1 lists all PhenoPlot elements that the user can choose for plotting depending on the features measured. Like other visualization tools, such as heatmaps and star and facial glyphs, data scaling is required in PhenoPlot. In the example shown (Fig. 1a), the cell body, nucleus, and perinuclear regions are represented using ellipses. The length and width of each of these objects are represented as the major and minor dimension of the ellipse, respectively. Dimensional variables (that is, length and width) should be scaled together to a 0.1–1 interval to maintain the aspect ratio between different dimensions and implicitly represent additional dimensions (for example, cell width-to-length ratio). In Fig. 1a, the number of nuclei is plotted as subcircles within the nuclear ellipse. The relative area of cell protrusions, such as lamellipodia, is represented on the top of the cell as a half-ellipse whose major dimension is proportional to the relative protrusion area (Fig. 1a). Intensities of the cell, nucleus and perinuclear regions are represented by mapping average intensity values of fluorescent markers to different colour hues.

To increase the number of dimensions that can be represented in PhenoPlot, we devised the concept of ‘Proportional Filling’ that exploits the principle of visual closure where humans can easily perceive the value of partially filled object^11^. Given a variable scaled between 0 and 1, we represent the feature using a glyph and the value by filling part of the glyph in proportion to the variable value with a specified symbol or colour. For example, if we measure the neighbour fraction (NF), that is, the fraction of the cell border that is in contact with other cells, then we can represent NF as the fraction of cell ellipse border that is thickened or overlaid by a symbol (Fig. 1a). Other representations include the proportion of the cell ellipse that is filled with a symbol, which can be used to represent cellular texture, the number of mitochondria or the number of vesicles (Fig. 1a). Similarly, the proportion of the nucleus ellipse filled with a symbol can be used to represent nuclear texture. We also added three organelle glyphs (ellipse, rectangle and line), where the height of the filled portion of the organelle is proportional to the variable value (Fig. 1a,b). These organelle glyphs can be used to represent an organelle intensity, quantity or texture. In total, eight features are provided that exploit proportional filling.

PhenoPlot allows the customization of different element colours and line styles and the specification of cell positions in a two-dimensional plane. Importantly, many PhenoPlot elements are colour independent, which increases its usability. A figure legend will be drawn automatically using the user input for feature names. Figure 1b shows the appearance of PhenoPlot elements representing different values for 15 variables (Supplementary Table 2). Unlike other visualization methods such as bar charts (Supplementary Fig. 1a), heatmaps (Supplementary Fig. 1b), star glyphs (Supplementary Fig. 1c and Supplementary Table 3) and Chernoff faces (Supplementary Fig. 1d and Supplementary Table 3), PhenoPlot represents particular cellular features intuitively (for example, cell shape, texture features or nuclear morphology).

### Profiling breast cancer cell lines morphology with PhenoPlot

**Figure 2:**
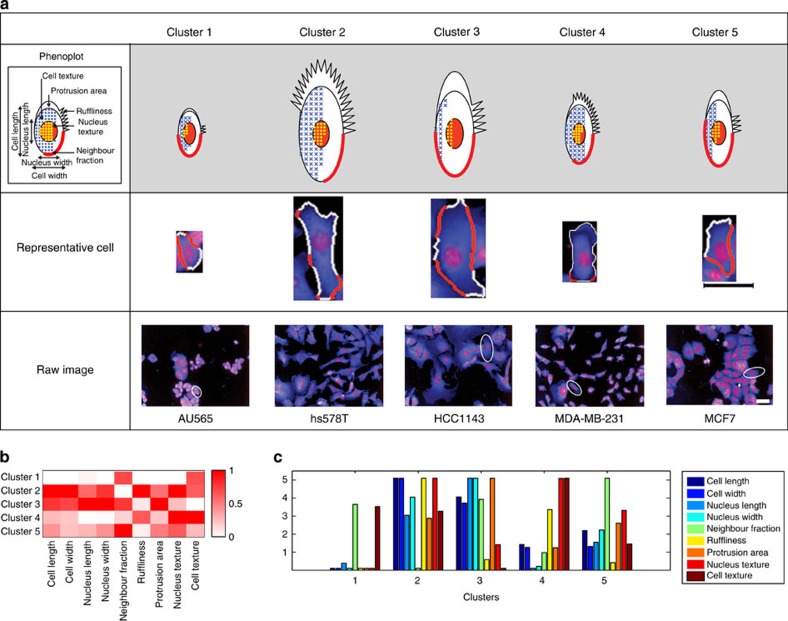
Visualization of morphological clusters of 19 breast cell lines. (**a**) Top row: PhenoPlot of the average of nine morphological features for each cluster. Middle row: representative image of a single cell from each cluster outlined in white and red, where red indicates the cell border in contact with other cells (NF). Scale bars, 50 μm. Bottom row: selected raw cell image from each cluster. Scale bars, 50 μm. (**b**) Heatmap of the average of nine morphological features for each cluster. (**c**) Bar chart of the average of nine morphological features for each cluster.

To demonstrate the utility of PhenoPlot, we generated PhenoPlots to describe the phenotypes of 19 breast cell lines, which are predominantly derived from human tumours (Supplementary Table 4). For each cell line, nuclear and cell bodies were fluorescently labelled, fixed, and imaged by confocal microscopy (Methods). Nine features were plotted for each cell including the length and the width of the cells and nuclei; the area of cellular protrusions; NF, which measures the fraction of cell border in contact with other cells; cellular ruffliness, which reflects the irregularity of the cell border; and the cellular and nuclear textures, which describe the distribution of pixel intensity in these regions (see Methods). Hierarchical clustering was used to group cell lines with similar morphologies into five clusters (Supplementary Fig. 2). We used PhenoPlot to visualize the average measurements for each cluster and produce intuitive representations based on the measurements of 155,811 cells (Fig. 2a, top row). Using PhenoPlot, we are able to better visualize aspects of cell morphology that are otherwise difficult for the human observer to appreciate. For example, the PhenoPlot of cells in cluster 1 shows that they are round, poorly spread, have high NF, low nuclear texture index and do not form protrusions (Fig. 2a). In contrast, the PhenoPlot of cells in cluster 2 shows that cells have extensive ruffles, low NF and high values of cellular and nuclear texture index. On the basis of the high value of protrusiveness, ruffliness and texture, we infer that the cells in cluster 2 are likely to be highly motile. This notion is consistent with the fact that hs578T and MDA-MB-157 cells are derived from metastatic breast cancer and are known to be invasive^12^. PhenoPlot shows that cells in cluster 3 are far less ruffly and textured and have higher NF than cells in cluster 2, suggesting that they are less motile. On the basis of their PhenoPlots, cells in cluster 4 appear to have an intermediate phenotype between clusters 1 and 2, while cells in cluster 5 seem to be similar to cells in cluster 3, but less spread. Thus, PhenoPlots provide effective and intuitive pictorial representations of cellular phenotypes that allow the interpretation of quantitative results and their relation to cellular images.

Discriminating between phenotypes of different clusters and making inferences regarding underlying biological process is challenging when using either images of a ‘representative cell’ (which is a cell with features closest to the average of all cells in the cluster), or images containing many cells (Fig. 2a middle and bottom rows and Supplementary Fig. 3). For example, cells in clusters 2 and 3 appear to have similar large, spread, flat shapes as determined by raw images (Supplementary Fig. 3), even though cells in cluster 2 exhibit far more ruffles than cells in cluster 3 (Fig. 2a top and b,c). Moreover, it is difficult for humans to appreciate from raw images that cluster 4 cells are the most ‘textured’ of all cells in the data set (Supplementary Fig. 3). It is also difficult for humans to appreciate the relationships between variables using raw images. For example, cluster 2 and 3 cells are both spread, but the ratio of ruffles to protrusiveness is very different (Fig. 2a top).

By comparison, typical visualization methods such as heatmaps or bar charts are not intuitive representations of phenotypes and are not easy to relate to cell images. For example, heatmaps represent the variables using colour shades of boxes (Fig. 2b), but these boxes do not reflect the visual appearance of the feature. Thus, it is difficult to picture how cells look from a heatmap, especially when many dimensions are displayed. Although bar charts are effective in identifying differences between the values of a few variables, it is difficult for the analyst to interpret a biological phenotype from this representation (Fig. 2c). Furthermore, it is difficult to understand the relationship between variables using heatmaps or bar charts, because features are compared individually.

### PhenoPlot is a flexible visualization method

**Figure 3:**
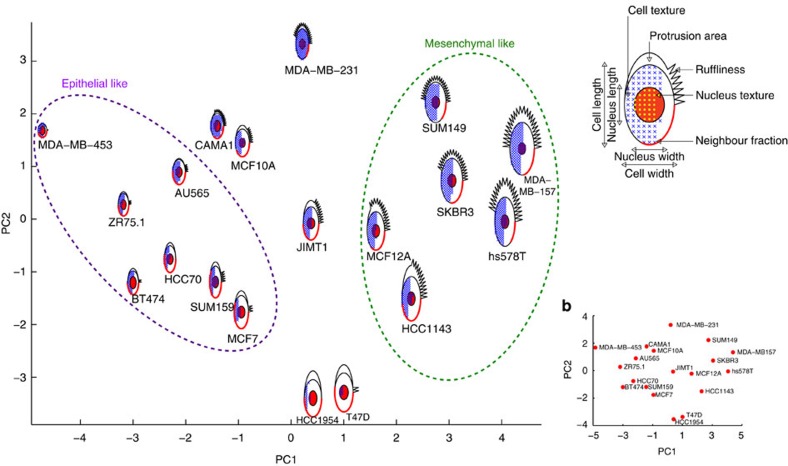
PhenoPlots projected in the first two PCs. (**a**) PhenoPlots of the average morphological measurements for 19 breast cell lines where the cell position in the two-dimensional plane is based on the first two PCs (PCA is applied to the same morphological measurements in Supplementary Fig. 2). (**b**) Average measurements of breast cell line morphology projected in the first two PCs.

Like other glyph-based approaches, PhenoPlots are independent of XY coordinates. This makes PhenoPlot a flexible tool that can be combined with other visualization methods. Furthermore, extra dimensions can be visualized using the position of PhenoPlots in a two-dimensional plane. For example, projecting PhenoPlots of average measurements for the different breast cell lines in the first two principal components (PCs) of the data facilitates the identification of phenotypic similarities and differences between cell lines (Fig. 3a). Figure 3a shows that cell lines on the left-hand side have epithelial-like shapes (low protrusiveness, less spread, and high NF), cells on the right-hand side have mesenchymal-like shapes (highly protrusive and ruffly, more spread, and low NF), while cell lines with intermediate morphologies are in the middle. Moreover, interesting relationships can be easily identified from this representation. For example, mesenchymal-like cell lines have higher nuclear texture than epithelial-like cell lines except for MCF10A and SUM159, and some of the cell lines with intermediate morphology have increased nuclear texture values. This observation can trigger further experiments to investigate the nature of nuclear texture differences between epithelial and mesenchymal phenotypes. Conversely, a typical scatter plot provides no information on the nature of differences between cell lines (Fig. 3b). Thus, PhenoPlot is a flexible method that can assist data analysis and identification of new hypotheses and complement other analysis and visualization techniques.

## Discussion

Visualization is essential for understanding and interpreting complex data extracted from cellular images. The currently available general-purpose visualization tools, such as heatmaps or parallel coordinates, are difficult to relate to biological phenomena. These methods are usually accompanied by qualitative examination of cellular images to identify cells representative of the quantitative phenotypes^13^,^14^, which is a tedious task that requires biological expertise, and is prone to bias. Many examples in the literature employ pictorial representations to explain biological phenotypes, but these examples are usually drawn manually and are generally not quantitative^15^,^16^,^17^. PhenoPlot formalizes a general pictorial representation of cells using various visual encodings and novel visualization techniques to concisely and quantitatively represent high-content data. PhenoPlot is available as a Matlab toolbox, which allows the integration of the visualization step with data exploration and data analysis steps. To increase PhenoPlot usability, we developed a simple GUI that can be easily used by biologists. We propose that PhenoPlot can facilitate exploration, understanding, memory and communication of cellular imaging data.

To maximize the effectiveness of PhenoPlot in communicating research results, visualization principles should be considered. These include the use of colour to make the most relevant features to the biological question more salient^18^, the application of the Gestalt principle of proximity^11^ by plotting cells in PC space and the removal of dimensions that do not convey information to the reader^19^.

‘An image is worth a thousand words’, but the challenge in high-content imaging is to summarize thousands of images in a few figures. To our knowledge, PhenoPlot is the first method that is specifically designed to represent cellular imaging data in an intuitive way so that it can be easily linked to the biological phenotype. This allows the effective visualization of multiple dimensions, which can reveal complex relationships that might otherwise be missed. Furthermore, PhenoPlot can aid the understanding and interpretation of quantitative results. Importantly, extensive biological expertise is not required to understand the visual elements in PhenoPlot, which make it a useful tool in science communication.

## Methods

### Implementation

The PhenoPlot toolbox was developed using Matlab 2012a. PhenoPlot includes a GUI. The source code of PhenoPlot, demo files and the data sets used in this manuscript are provided in the Supplementary Software at http://www.icr.ac.uk/our-research/researchers-and-teams/dr-chris-bakal/resources.

### Experimental methods for breast cancer lines data set

MCF10A breast epithelial and AU565 breast tumour cells were obtained from ATCC (LCG Standards). MDAMB231, HCC70, HCC1143, HCC1954, MCF7, T47D, BT474, CAMA1, MDAMB453 and hs578T breast tumour and MCF12A non-tumour cells were obtained from the laboratory of Alan Ashworth (Breakthrough Breast Cancer, ICR). SUM149, SUM159, MDAMB157, JIMT1, SKBR3 and ZR75.1 breast tumour cells were obtained from the laboratory of Jorge Reis-Filho (Breakthrough Breast Cancer, ICR).

All cell lines were cultured in DMEM:F12 Glutamax medium supplemented with 5% heat-inactivated fetal bovine serum, unless otherwise indicated.

Cells were seeded in 384-well plates at concentrations ranging from 1,000 to 3,000 cells per well, depending on the size and proliferation rate of the cell line. Cells were fixed on day 3 after plating. Before fixation, 10 μM dihydroethidium (2-hydroethidium, dihydroethidium (DHE); Invitrogen) was added to all wells. Cells were fixed with 4% formaldehyde at room temperature for 10 min, washed with PBS and permeabilized with PBS/0.1% Triton-X-100 for 10 min at room temperature. Nuclear DNA was stained with 4′,6-diamino-2-phenylindole (DAPI; Sigma). Sequential image acquisition was performed with a × 20 air objective using an automated spinning disc confocal microscope, the Opera HCS (PerkinElmer). Fourteen wells and 12 fields per well were imaged per condition.

### Image processing

Customized image analysis scripts were developed and applied using Acapella Studio 2.7 (PerkinElmer).

Nuclei were detected using the DAPI channel, and the cytoplasm was detected based on the DHE channel using the nucleus object as a seed. Cell and nucleus length, width and area and NF were extracted using Acapella functions. Twenty texture features (eight SER, eight Gabor^20^ and four Haralick^21^) were calculated for both the DAPI channel in the nuclei region and the DHE channel in the cell core region. Protrusions were calculated as follows. For each cell, the average pixel intensity in the DHE channel was calculated and the largest subobject with more than 70% of the cell average intensity was selected as the cell core and the rest of the cell was selected as protrusion area. Ruffliness was calculated as an SER Edge texture feature in the cell membrane divided by the cell form factor, which we found to be representative of cell edge ruffling. In total, 52 features were extracted. Mitotic cells (cells with high DAPI intensity judged on experiment and cell line bases), small objects and border objects were filtered out.

### Computational analysis

All computational analysis steps were performed using Matlab.

### Feature transformation

To obtain a texture features index, we scaled texture features and then applied PCA to the nucleus and cell texture features individually. We used the first PC from each set as compressed texture index.

For the hierarchical clustering and PCA, standard normalization was used so that all features were on the same scale. Euclidean distance and average linkage were used for the hierarchical clustering.

To generate the PhenoPlots in Figs 2a and 3a, dimensional features (cell length and width, and nucleus length and width) were scaled together to a 0.1–1 interval (to maintain aspect ratio) by subtracting the minimum of all dimensional features and dividing by the range of all dimensional features, and then multiplying the result by 0.9 and adding 0.1. The scaling for dimensional features start at 0.1 to avoid losing an object when it has relatively the lowest length or width. All other features were scaled between 0 and 1. Supplementary Table 5 lists the features used for the represented elements.

## Author contributions

H.S. conceived the study, designed and developed the tool and performed the image and data analysis. J.E.S. performed the breast cell lines experiment. H.S. and C.B. wrote the manuscript.

## Additional information

**How to cite this article:** Sailem, H. *et al.* Visualizing cellular imaging data using PhenoPlot. *Nat. Commun.* 6:5825 doi: 10.1038/ncomms6825 (2015).

## Supplementary Material

Supplementary Figures, Supplementary Tables and Supplementary Software Supplementary Figures 1-3 and Supplementary Tables 1-5

Supplementary Software PhenoPlot is a Matlab toolbox with an interactive Graphical User Interface (GUI) that generates cell-like glyphs from imaging data. The software requires Matlab 2012 and is best-used with data extracted from cellular images, but can be used to represent any numerical data. A guide on using the software both from the command line, and using the GUI, is provided in the file PhenoPlot_manual.pdf.
