# Supplementary material for: Visualizing cellular imaging data using PhenoPlot
Source: Nat Commun. 2015 Jan 8;6:5825. doi: 10.1038/ncomms6825 (PMC4354266; doi:10.1038/ncomms6825)
Supplement: Supplementary Figures, Supplementary Tables and Supplementary Software — Supplementary Figures 1-3 and Supplementary Tables 1-5 [file ncomms6825-s1.pdf]

Supplementary Figures

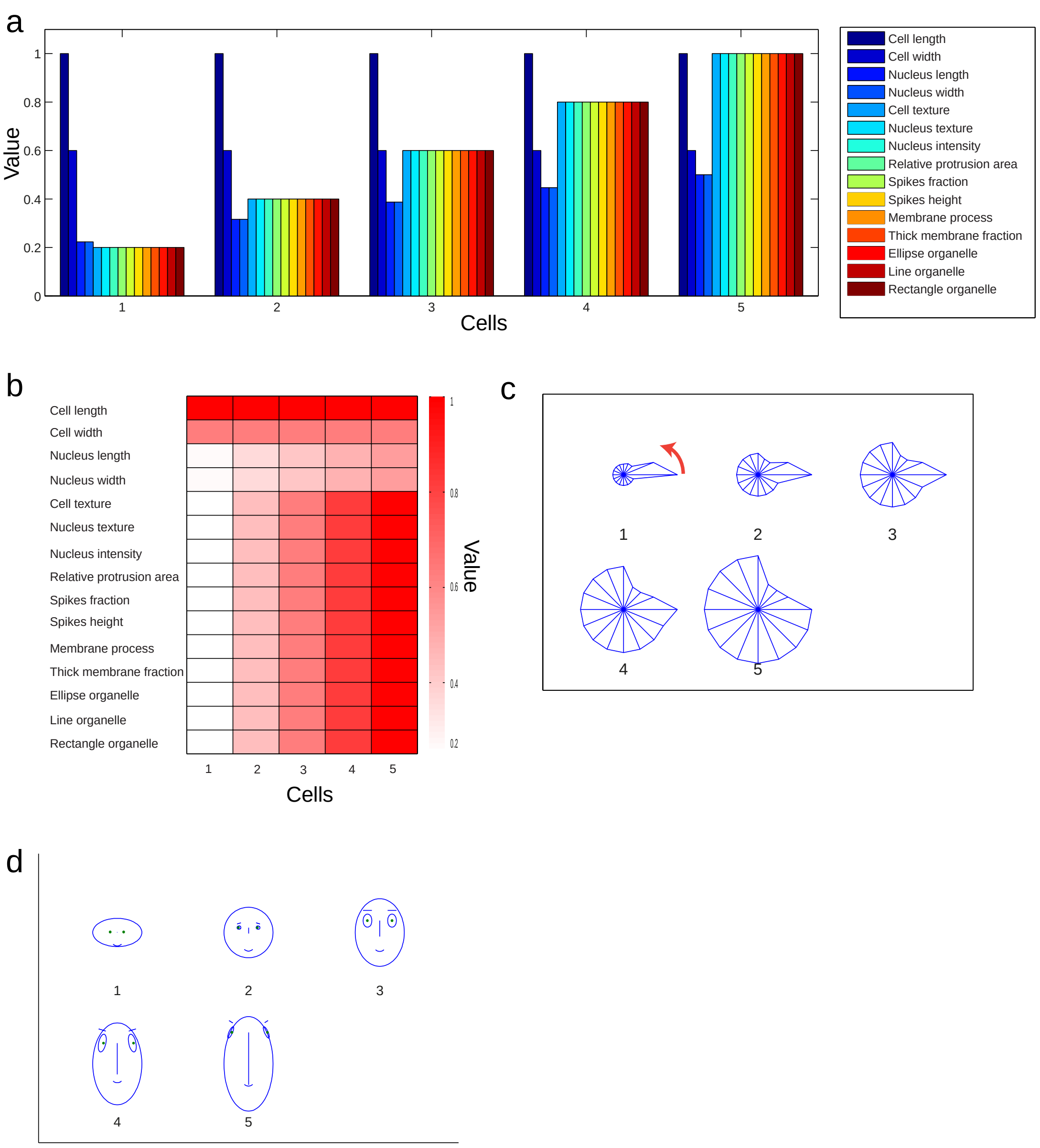

Supplementary Figure 1: Representation of traditional visualization methods. The representations are generated for the data in Figure 1b. (a) Bar chart representation. (b) Heat map. (c) Star glyphs. Legend is shown in Supplementary Table 3 where features are listed in order of their placement in the glyph starting from the red arrow and moving in a counter-clockwise direction. d) Chernoff faces, (e) Legend for star glyphs. Legend is shown in Supplementary Table 3.

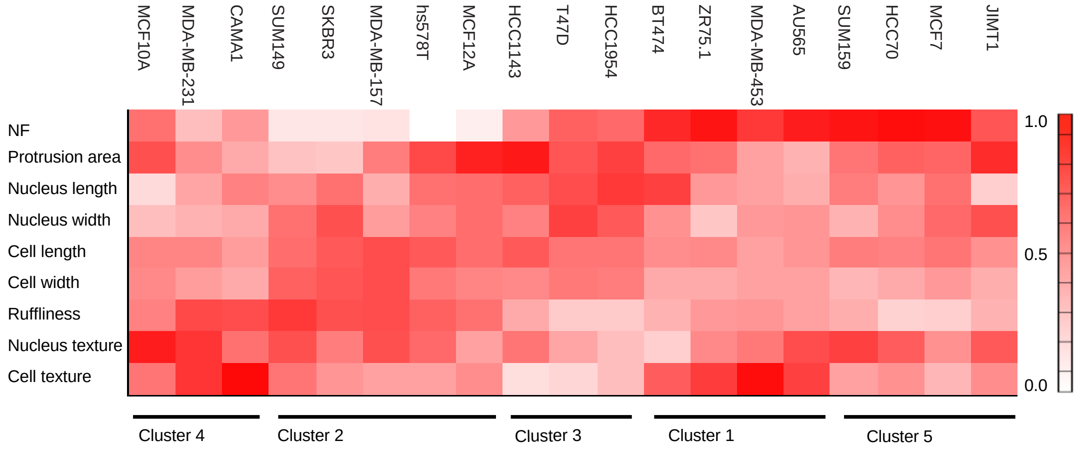

Supplementary Figure 2: Clustering of breast cell lines average measurements.

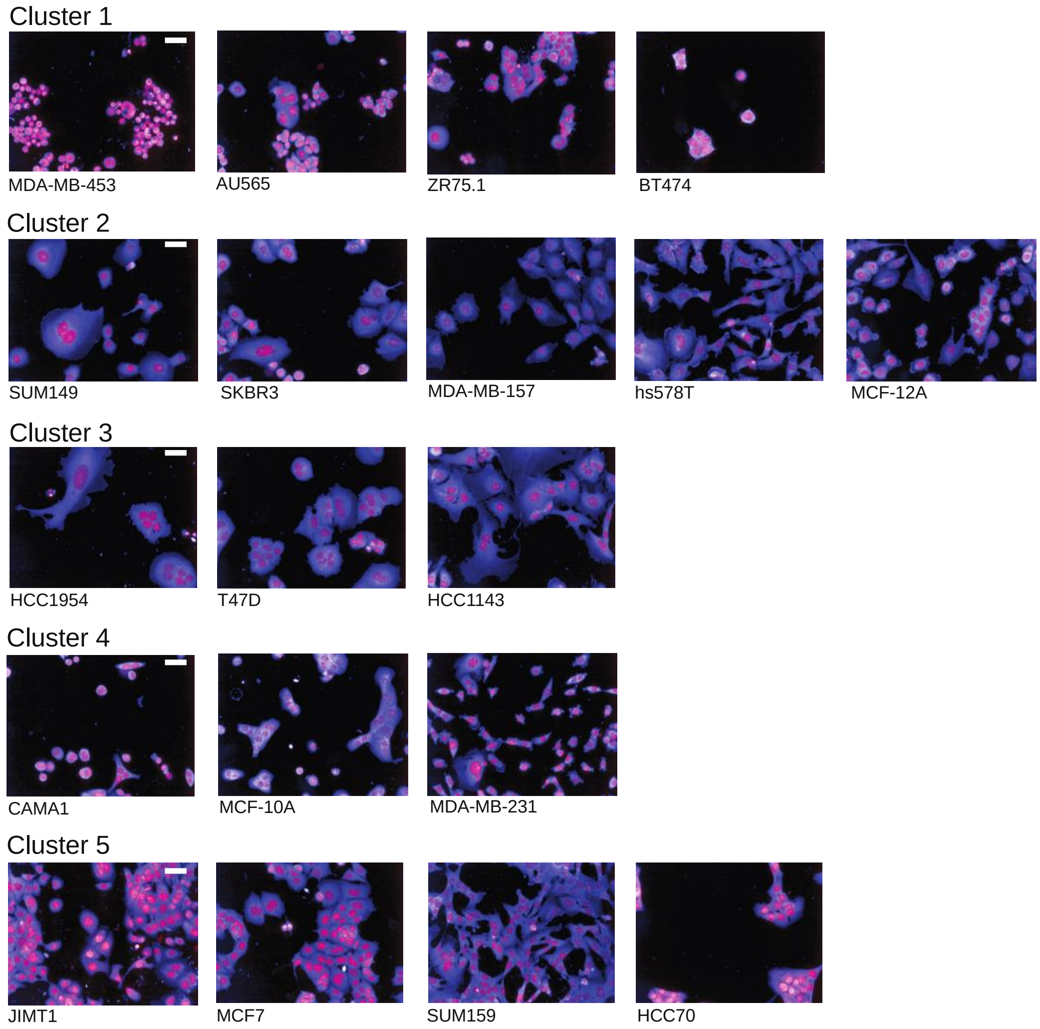

Supplementary Figure 3: Overlay images of breast cell lines. DAPI (nuclei) is shown in red and DHE (cytoplasm) in blue. Scale bars = 50 µm.

Supplementary Tables

Supplementary Table 1: Available elements in PhenoPlot

Element	Description
Main ellipse (dimensions)	This is the only mandatory element. Requires length (ellipse major dimension) and width (ellipse minor dimension). The color of the main ellipse border can be changed.
Main ellipse color	This element specifies the color of *main ellipses*. This can be fixed; the same for all ellipses, or variable; each ellipse can have different color depending on the feature value.
Inner sub-ellipse	Similar to the *main ellipse* element. The *inner sub-ellipse* dimensions should be less than the *main ellipse* dimensions. This can represent the nuclei or sub-cellular organelles.
Inner sub-ellipse color	Similar to the *main ellipse color* element but for the *inner sub-ellipse*.
Sub-circles	Draws *X* circles inside the inner *sub-ellipse* using line specification. If *X* is a natural number then *X* full circles will be drawn. If X is a rational number, then the last sub-circle will be drawn as circular sector where its arc length is proportional to the decimal part of *X*. This element can represent either multi-nucleate cells, number of micronuclei or number of nucleoli.
Outer sub-ellipse	Similar to the *main ellipse* element.
Relative protrusion area	The protrusion is drawn as a half ellipse on the top of the *main ellipse* and with the same center as the *main ellipse*. The protrusion ellipse minor dimension is the same as the main ellipse, while the major diameter is calculated as in the equation:<br>$Y=X+X*Z$<br>Where *Y* is the Protrusion ellipse major dimension, *X* is the *main ellipse* major dimension, and *Z* is the relative protrusion area.
Spikes	Draws a fraction of the *main ellipse* border in a jagged line where the length of the jagged fraction is proportional to the variable value. If the *relative protrusion area* element is used then it draws a fraction of the protrusion half ellipse border in a jagged line. The height of the jagged border is proportional to the spike height. The line style and color will be the same as the *main ellipse*.
Thick Membrane fraction	Draws a fraction of the *main ellipse* border in a thick line using the specified color.
Membrane process	Overlays a fraction of the *main ellipse* border using a specified symbol where the length of the overlaid fraction is proportional to the feature value.
Main ellipse filling	Generates *X* points distributed equally throughout the *main ellipse* using ellipse_grid function at http://people.sc.fsu.edu/~jburkardt/c_src/ellipse_grid/ellipse_grid.html. Draws a fraction of these points using the symbol and color specified based on the feature value. This can represent features that describe texture, number of vesicles or mitochondria.
Inner sub-ellipse filling	Similar to the *Main ellipse filling* element but for the *inner sub-ellipse*.
Ellipse, rectangle, or line organelle	Draws a glyph inside the *main ellipse* and fills part of the drawn glyph with the specified color where the height of the filled part is proportional to the feature value. The size of the glyph is proportional to the *main*

	ellipse minor dimension. The ellipse organelle is drawn on the top left of the cell, line organelle on the top right and the rectangle ellipse on the bottom right.

Supplementary Table 2: Feature values used to represent the elements in Fig. 1b

Element	Feature	Feature value
Main ellipse	Cell length	{1,1,1,1,1}
	Cell width	{0.6,0.6,0.6,0.6,0.6}
Inner sub-ellipse	Nucleus length	{0.22,0.31,0.38, 0.44, 0.5}
	Nucleus width	{0.22,0.31,0.38, 0.44, 0.5}
Main ellipse filling	Cell texture	{0.2,0.4,0.6,0.8,1.0}
Inner sub-ellipse filling	Nucleus texture	{0.2,0.4,0.6,0.8,1.0}
Inner sub-ellipse color	Nucleus intensity	{0.2,0.4,0.6,0.8,1.0}
Relative protrusion area	Relative protrusion area	{0.1,0.2,0.3,0.4,0.5}
Spikes	Spikes fraction	{0.2,0.4,0.6,0.8,1.0}
	Spikes height	{0.2,0.4,0.6,0.8,1.0}
Membrane process	Same as element name	{0.2,0.4,0.6,0.8,1.0}
Thick membrane fraction	Same as element name	{0.2,0.4,0.6,0.8,1.0}
Ellipse organelle, line organelle, and rectangle organelle	Same as element name	{0.2,0.4,0.6,0.8,1.0}

Supplementary Table 3: Legend for Supplementary Figure 1c-d

Face element	Feature represented
Size of face	Cell length
Forehead/jaw relative arc length	Cell width
Shape of forehead	Nucleus length
Shape of jaw	Nucleus width
Width between eyes	Cell texture
Vertical position of eyes	Nucleus texture
Height of eyes	Nucleus intensity
Width of eyes (this also affects eyebrow width)	Relative protrusion area
Angle of eyes (this also affects eyebrow angle)	Spikes fraction
Vertical position of eyebrows	Spikes height
Width of eyebrows (relative to eyes)	Membrane process
Angle of eyebrows (relative to eyes)	Thick membrane fraction
Direction of pupils	Ellipse organelle
Length of nose	Line organelle
Vertical position of mouth	Rectangle organelle
Shape of mouth	No value
Mouth arc length	No value

Supplementary Table 4: List of breast cell lines and their classification

Cell line	Molecular class
MCF10A*	BasalB
MCF12A*	BasalB
hs578T	BasalB
MDA-MB-157	BasalB
MDA-MB-231	BasalB
JIMT1	BasalB
SUM149	BasalB
SUM159	BasalB
HCC1954	BasalA
HCC70	BasalA
HCC1143	BasalA
AU565	Luminal
ZR75.1	Luminal
CAMA1	Luminal
BT474	Luminal
SKBR3	Luminal
T47D	Luminal
MCF7	Luminal
MDA-MB-453	Luminal

* Non tumor.

Supplementary Table 5: Elements and features used to represent the elements in Fig. 2a and 3a

Element	Feature
Main ellipse	Cell length
	Cell width
Main ellipse filling	Cell texture
Inner sub-ellipse	Nucleus length
	Nucleus width
Inner sub-ellipse filling	Nucleus texture
Relative protrusion value	Relative protrusion area
Thick membrane fraction	NF
Spikes	Ruffliness
