# Supplementary material for: Visualizing cellular imaging data using PhenoPlot
Source: Nat Commun. 2015 Jan 8;6:5825. doi: 10.1038/ncomms6825 (PMC4354266; doi:10.1038/ncomms6825)
Supplement: Supplementary Software — PhenoPlot is a Matlab toolbox with an interactive Graphical User Interface (GUI) that generates cell-like glyphs from imaging data. The software requires Matlab 2012 and is best-used with data extracted from cellular images, but can be used to represent any numerical data. A guide on using the software both from the command line, and using the GUI, is provided in the file PhenoPlot_manual.pdf. [file ncomms6825-s2.zip › Supplementary software/PhenoPlot/PhenoPlot_manual.pdf]

PhenoPlot Manual

By Heba Sailem
4/11/2014

Table of contents

I. Introduction 3
II. PhenoPlot requirements 3
III. Setup 3
IV. Data format 4
V. PhenoPlot elements and user interface 5
VI. How to use 8
VII. Demo 1: The morphology of breast cell lines clusters. 9
VIII. Demo 2: Representing various cellular markers 21
IX. Using PhenoPlot from the command line 23
X. Citing PhenoPlot 23
XI. Copyright 23

I. Introduction

This is a Matlab toolbox for visualizing cellular imaging data. PhenoPlot toolbox draws cell-like glyphs from multivariate data. The toolbox can be used to represent any numerical data. The toolbox is associated with the paper "Visualizing cellular imaging data using PhenoPlot" (Sailem et. al., 2014, Nature Communications). PhenoPlot can be invoked in the command line using PhenoPlot function or as Graphical User Interface (GUI) using PhenoPlot_gui.

II. PhenoPlot requirements

Matlab environment is required for PhenoPlot. PhenoPlot does not require special Matlab toolboxes, however figures are best viewed when the figure window is maximized and when saved to .eps or .PDF format. 'Export_fig' function from Matlab File Exchange can be used to export figures to PDF. This function is included in the toolbox. However it requires Ghostscript (download for Mac or Windows).

III. Setup

1. Download PhenoPlot package at

http://www.icr.ac.uk/our-research/researchers-and-teams/dr-chris-bakal/resources

2. Unzip PhenoPlot.zip to '\PhenoPlot' to a location of your choice.
3. To add '\PhenoPlot' to the search path of Matlab:
   - Open Matlab
   - Select the 'File' menu in Matlab and choose 'Set path'
   - Click on 'Add with subfolders' button and locate '\PhenoPlot' then click 'open' button.
   - Click 'Save' button.

4. Start PhenoPlot GUI by typing

```
>> PhenoPlot_gui
```

in the command window in Matlab and pressing the 'Enter' key.

IV. Data format

The software accepts data in a comma-separate values (csv) format. Columns should represent features/variables and rows should represent conditions or samples, see Table 1. Data should have a column header indicating feature names (shaded blue in Table 1) and row header indicating condition names (shaded grey in Table 1).

PhenoPlot provides an option to transform the data. If you choose to use your own normalization then all features should be scaled between 0.0 and 1.0 except for dimensional data, i.e. main ellipse length, main ellipse width, sub-ellipse length, and sub-ellipse width. Dimensional data should be scaled between 0.1 and 1.0 to avoid diminishing of the smallest object. They also should be scaled together so that main ellipse dimensions are kept in proportion with sub-ellipse dimensions. Alternatively you can ask PhenoPlot to transform your data by checking the 'Transform data' option from the GUI.

Table 1: Example data to be used in PhenoPlot

Condition	Cell Length	Cell Width	Number of nuclei	Nucleus length	Nucleus width
control	0.5	0.4	2	0.4	0.3
condition1	0.9	0.5	4	0.5	0.35
condition2	0.6	0.45	5	0.3	0.3
condition3	0.8	0.5	7	0.5	0.45
condition4	0.4	0.2	6	0.2	0.2

V. PhenoPlot elements and user interface

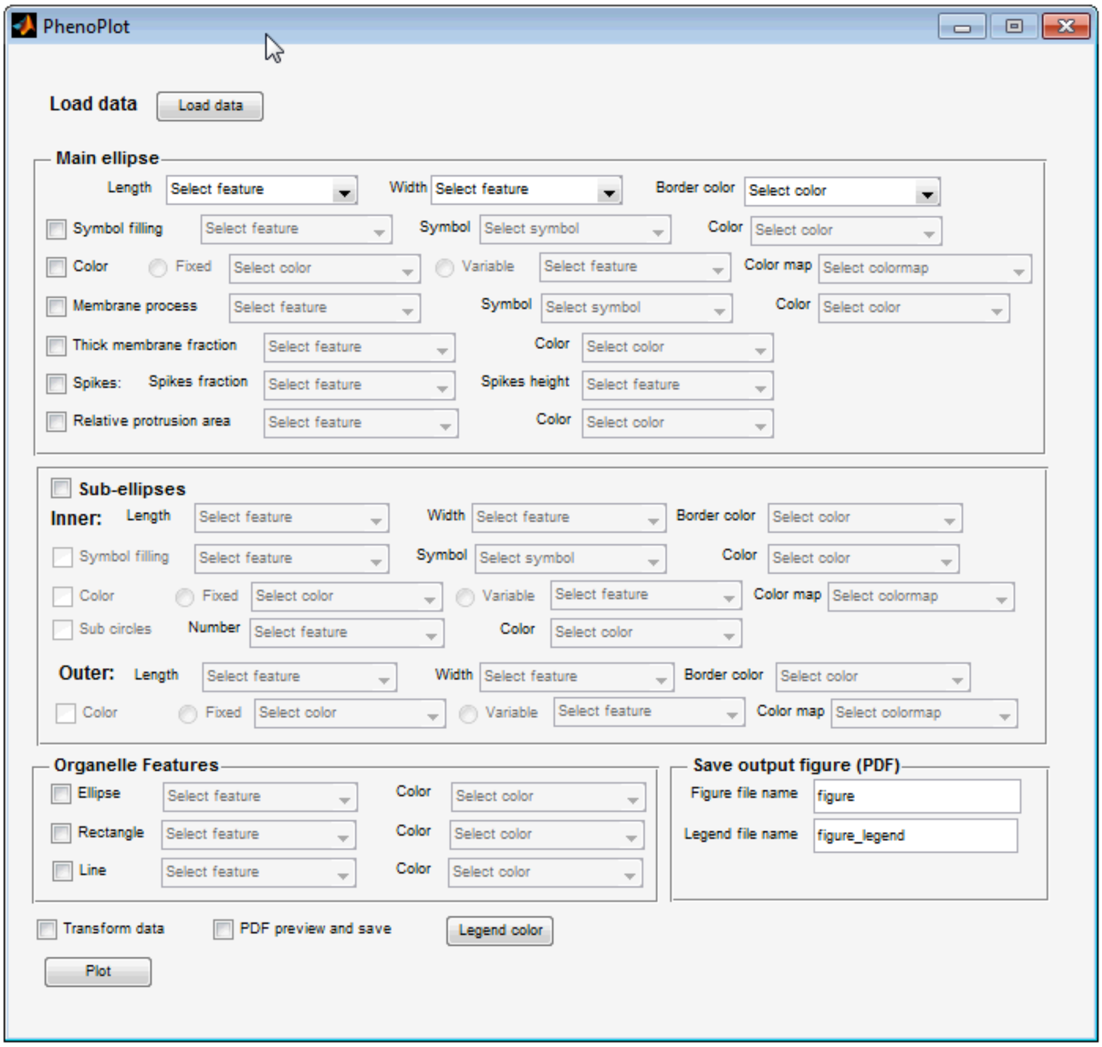

Figure 1: PhenoPlot GUI window

PhenoPlot window is composed of four panels (Figure 1):

- **Main ellipse panel**: the main ellipse panel is to specify the features and elements associated with this ellipse (Figure 2). The main ellipse can represent a cell, endoplasmic reticulum, or even a tumor.

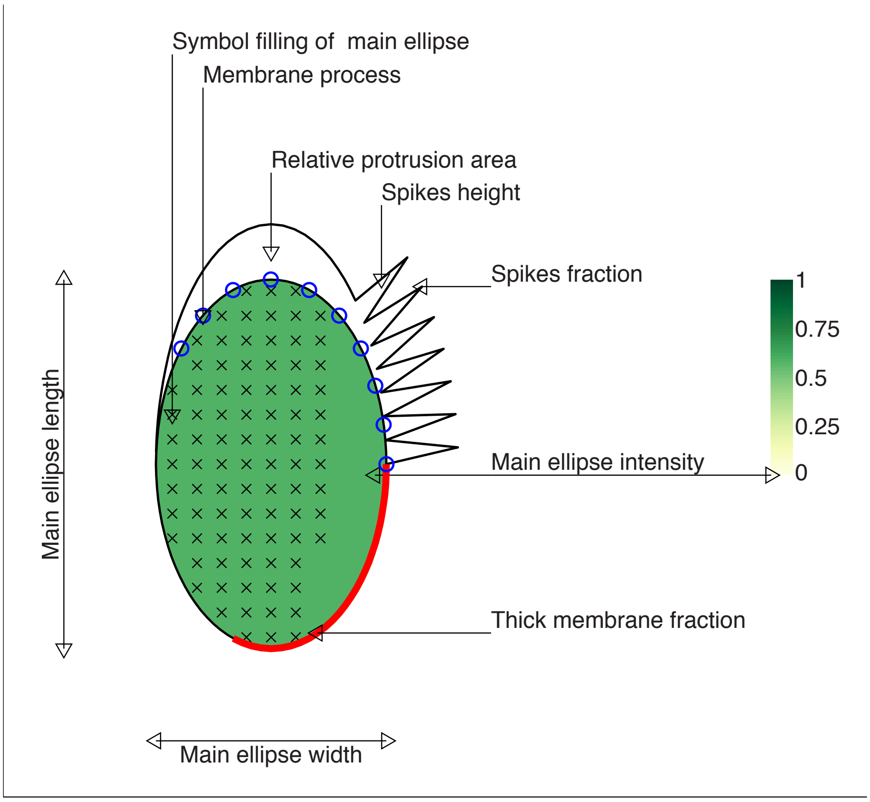

Figure 2: Elements of the main ellipse

- **Sub-ellipses panel**: sub-ellipses should have smaller dimensions than the main ellipse. Two sub-ellipses can be drawn: Inner and outer (Figure 3). The inner sub-ellipse has more options and could represent the nuclei (Figure 4) while the outer sub-ellipse can represent the endoplasmic reticulum or golgi bodies measurements. The elements in this panel are disabled until the 'Sub-ellipses' check box is ticked.

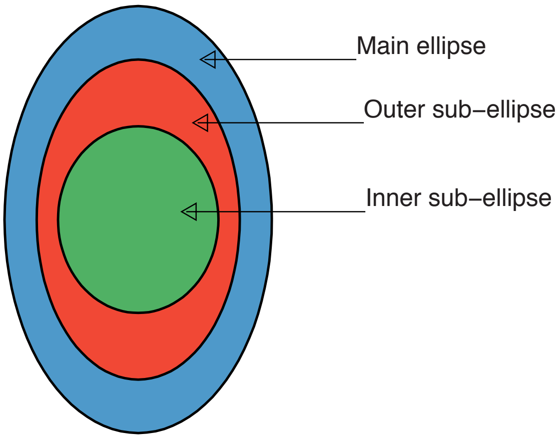

Figure 3: Main and sub-ellipses (inner and outer)

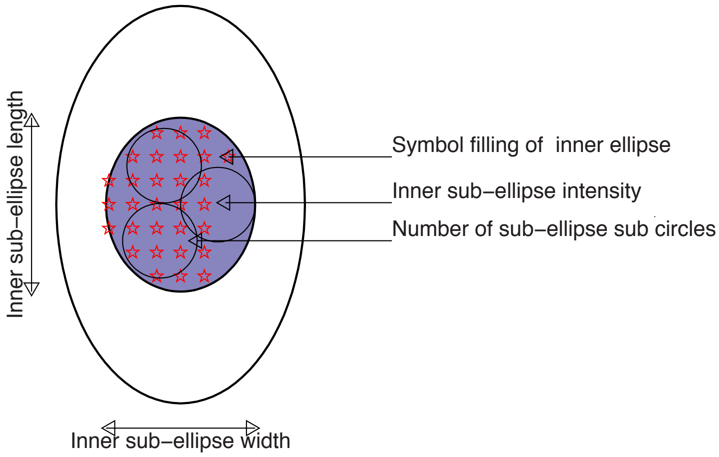

Figure 4: Elements of inner sub-ellipse

- **Organelles panel**: Organelle glyphs will be represented as small shapes around the nuclei and will be filled with the specified color depending on the variable value (Proportional Filling) (Figure 5).

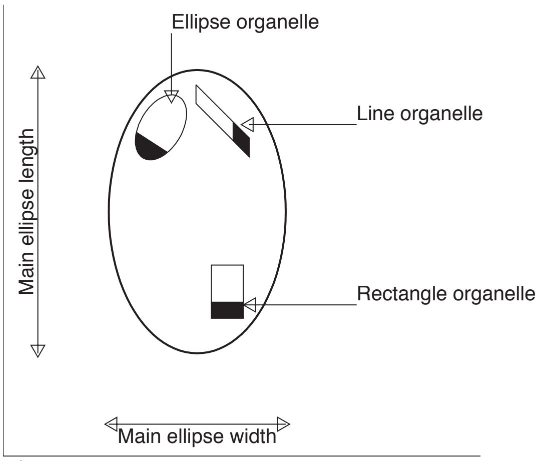

Figure 5: The organelle elements

- **Output panel**: this panel is for specifying the output figure and the legend figure file names. These will only be used if the 'PDF preview and save' check box is ticked (requires GhostScript to be installed, see "Setup").

VI. How to use

To plot your data use the following steps:

1- Click ‘Load data’ button to load data in PhenoPlot.
2- Locate your data or select one of the demo data files (e.g. demodata.csv)
3- The feature names in the column header will be loaded in the PhenoPlot GUI so that you can select a feature for each element.
4- Select the features to be plotted as main ellipse ‘length’ and ‘width’. Change the main ellipse ‘border color’ if needed. This is the only mandatory element you have to specify.
5- Check the other elements you want to plot. For each element you need to check that element to enable feature selection and then select the feature(s) to be plotted for that element.
6- When finished click ‘Plot’ button to generate the figure. For each figure the corresponding legend will be plotted in a separate figure so the user can scale the legend PhenoPlot separately. Elements used in plotting will be labeled in the legend figure using the feature names in the column header of the loaded csv file.

Optional

- Output figure is best viewed in PDF or ‘eps’ format. To view the resulting figure in PDF check ‘Save and preview’ option. The figure and the legend file will be saved to the current working folder. You can change the figure file name and the legend file name in ‘Save output figure’ panel (requires Ghostscript).
- Check ‘transform data’ to transform your data.
- You can change the color of legend arrows and text by clicking ‘Legend color’ button. This will open a color selection window. Select a color and press ‘OK’ button.

VII. Demo 1: The morphology of breast cell lines clusters.

This example will take you through the steps for plotting a dataset that describes the morphology of five Breast Cancer cell Lines (BCL) groups. The data is available in *‘BCL_clusters.csv’*.

1- Download and install PhenoPlot toolbox as in Setup section.
2- Type ‘PhenoPlot_gui’ in Matlab command line and press enter
3- Click on the ‘Load data’ button (Figure 6).
4- Locate BCL_clusters.csv file from PhenoPlot folder. The feature names in BCL_clusters.csv will be loaded in the PhenoPlot GUI so that you can select a feature for each element.

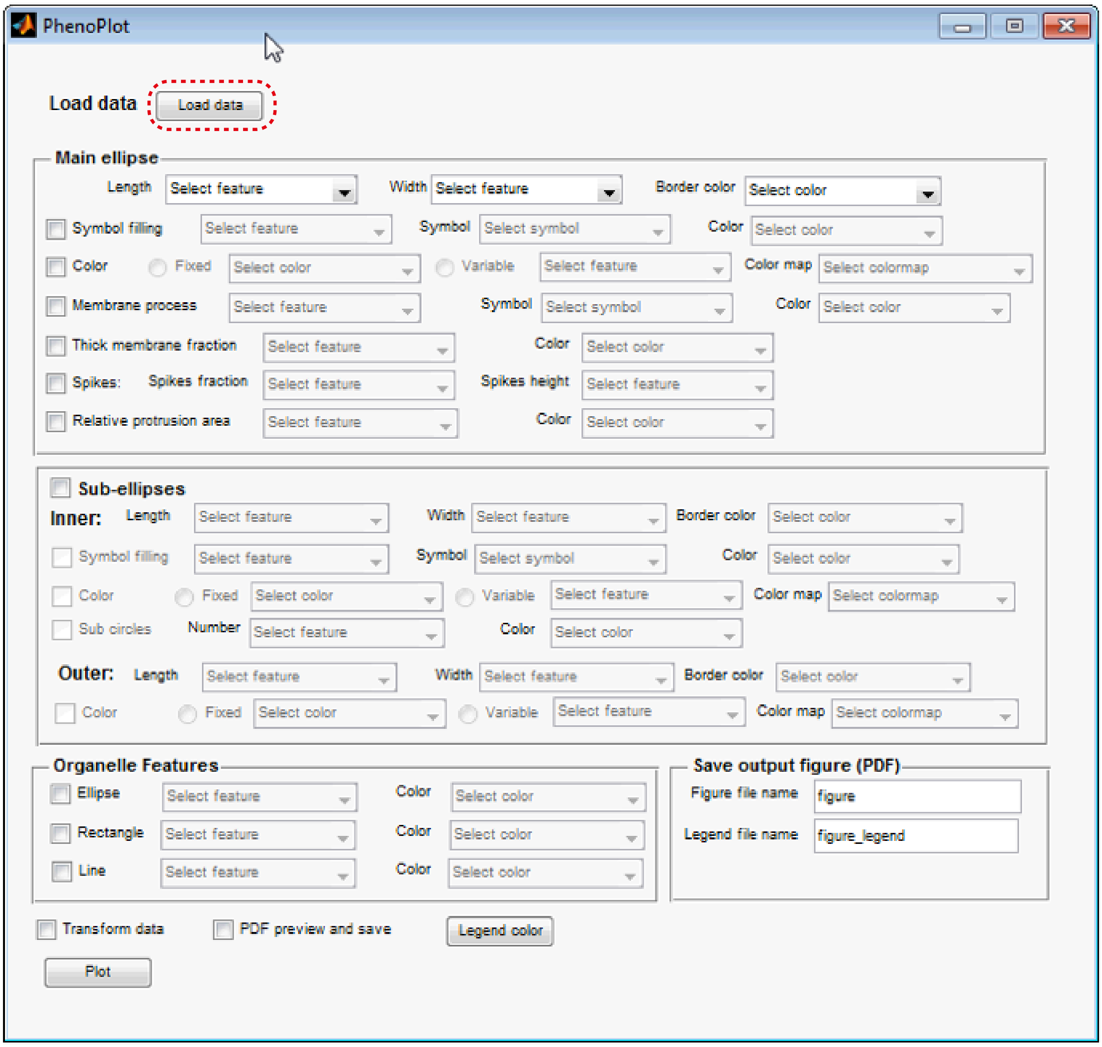

Figure 6: Loading data in PhenoPlot

5- First, we will represent the cellular dimensions using the 'Main ellipse' element in Phenoplot. In the main ellipse panel select Cell length from 'Length' drop-down list (Figure 7).

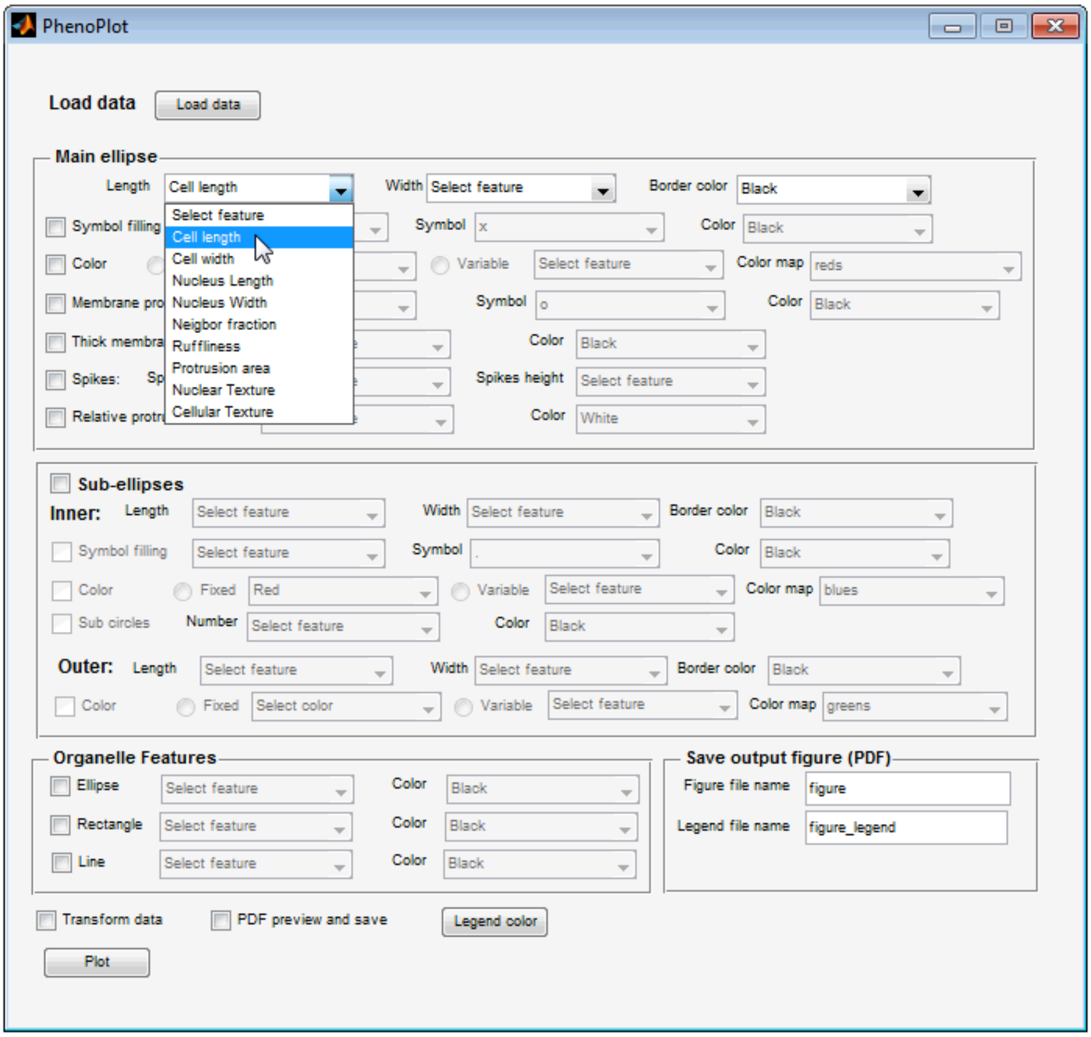

Figure 7: Specifying the length of the main ellipse

6- Similarly select Cell width from the ‘Width’ drop-down list (Figure 8).

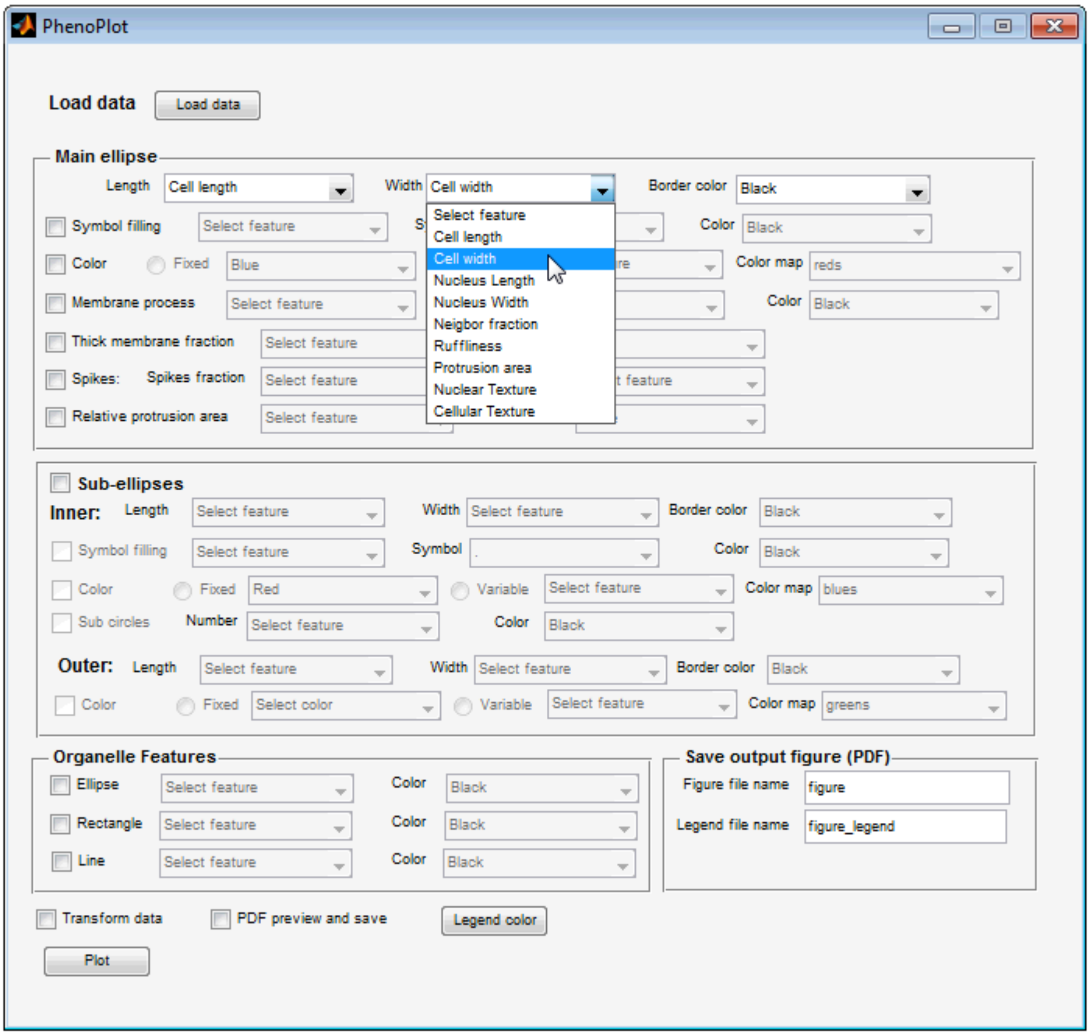

Figure 8: Specifying the width of the main ellipse

7- Choose Magenta from the ‘Border color’ drop-down list (Figure 9).

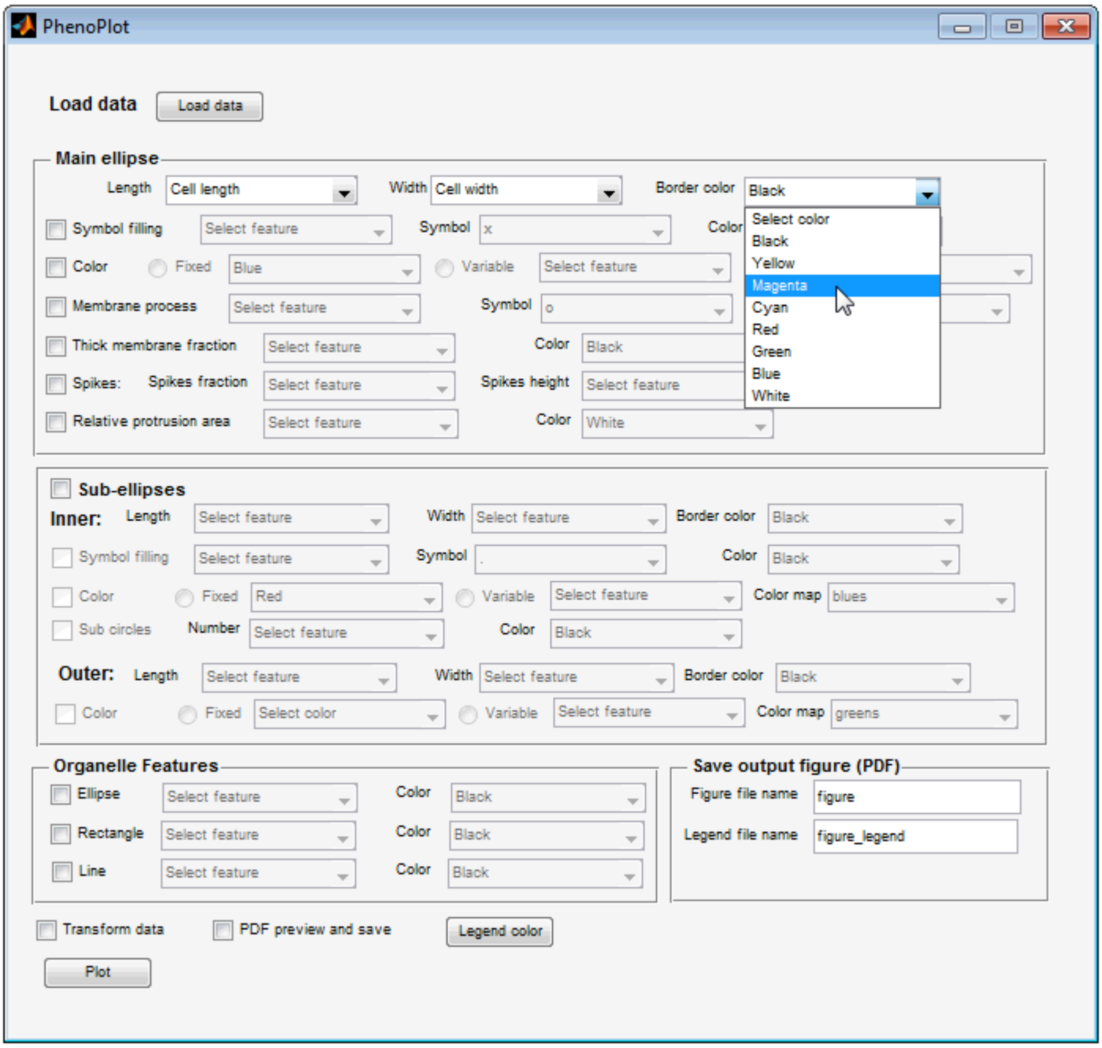

Figure 9: Specifying the border color of the main ellipse

8- Next we will fill the main ellipse with ‘x’ symbols depending on the value of cell texture. Cell texture is a measure of variation of the intensity in the cell (such as peaks and valleys of intensity).

   a. Check the ‘Symbol filling’ check box in the main ellipse panel (Figure 10).

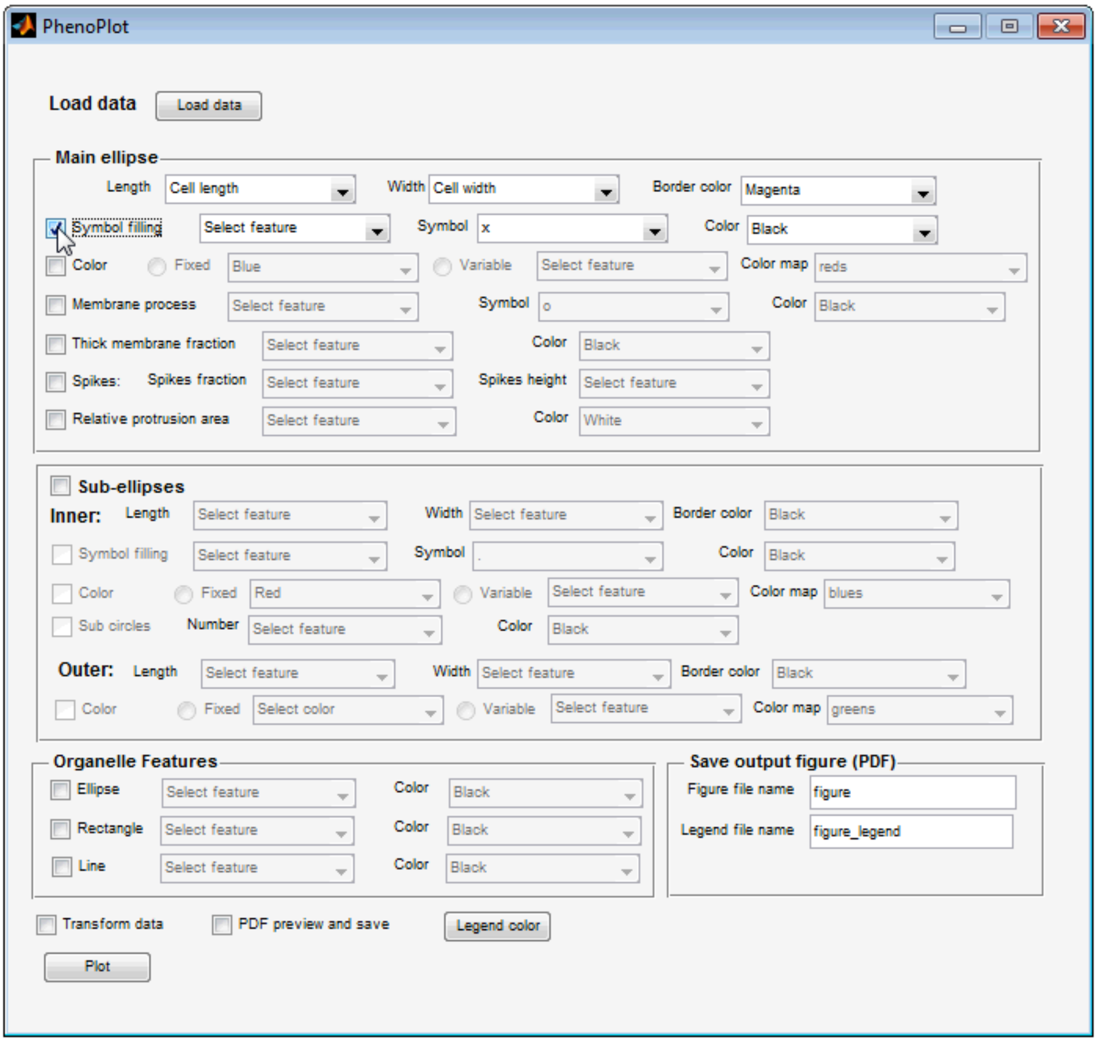

Figure 10: Selecting symbol filling element

b. This will enable the 'Symbol filling' drop-down list to allow assignment of the feature for this element. Select Cellular texture feature from the drop-down list (Figure 11).

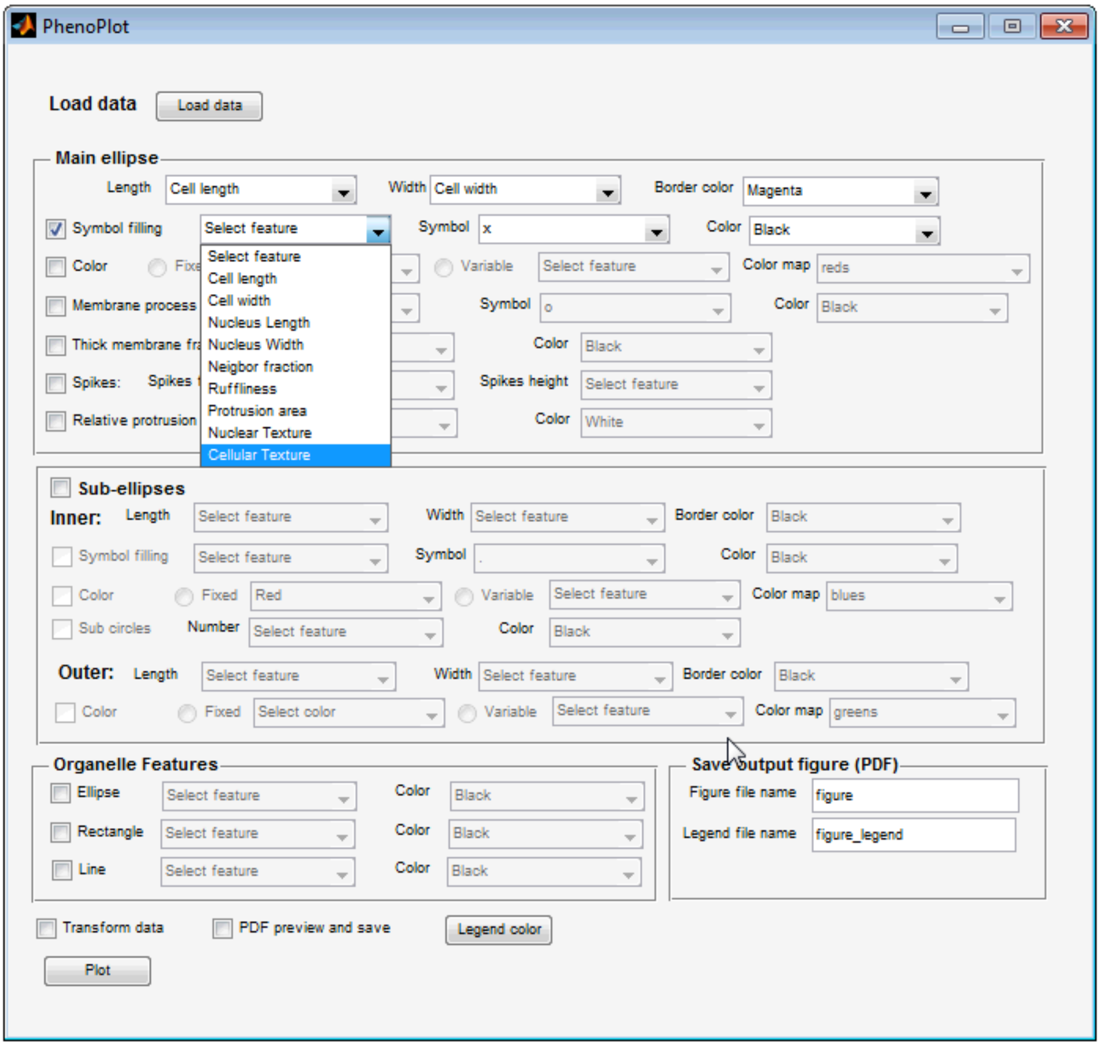

Figure 11: Choosing a feature for symbol filling of the Main ellipse

9- In the same way as outlined above, select other elements in the main ellipse panel as shown in Figure 12 as following:

   a. Check the ‘Thick membrane fraction’ check box and select Neighbor fraction feature from the corresponding drop-down list. This will be displayed as fraction of cell ellipse in thick border. Then change the ‘Color’ of ‘Thick membrane fraction’ option to magenta.

   b. Check the ‘Spikes’ check box. This element can represent two features the spikes fraction that can be representative of cell border irregularity, and the height of spikes. The second variable is optional. In this example we will use only the spikes fraction to represent cell ruffliness. Select the Ruffliness feature from the ‘Spikes fraction’ drop-down list.

   c. Check the ‘Relative protrusion area’ check box and select protrusion area feature from the corresponding drop-down list. This element will be drawn as a half ellipse on the top of the main ellipse with height proportional to its value.

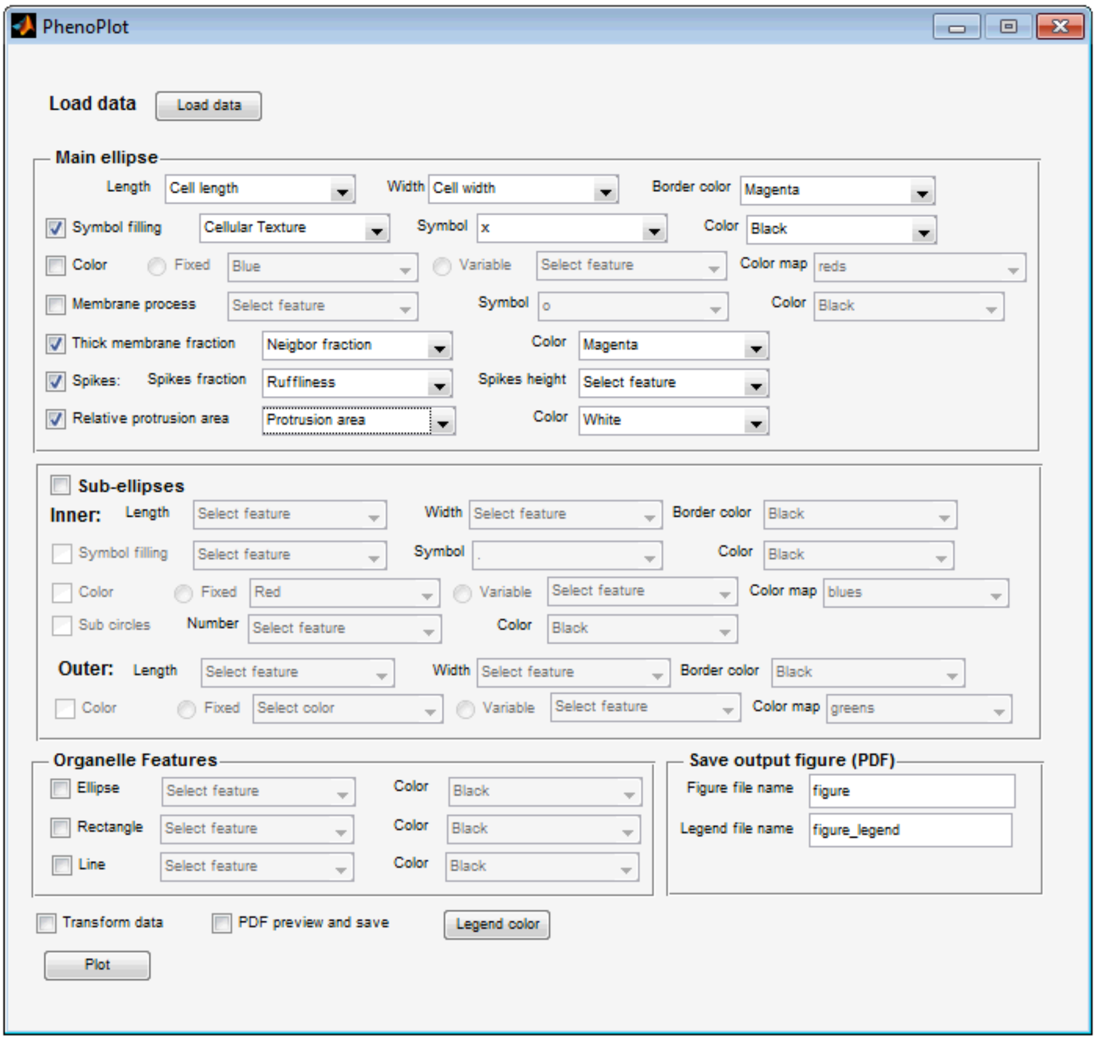

Figure 12: Selecting other elements in the Main ellipse panel

10- Next we will use the inner sub-ellipse element to represent nuclear measurements. Check the ‘Sub-ellipses’ check box to enable elements selection in this panel. Then select the features as shown in Figure 13 as following:

   d. Select the nucleus length feature from ‘Length’ drop-down list and nucleus width feature from ‘Width’ drop-down list.
   e. Next we will fill the inner sub-ellipse with dots (‘.’) depending on the value of nuclear texture. Nuclear texture is a measure of variation in intensity in the DAPI channel. Check the ‘Symbol filling’ check box in the sub-ellipse panel and then select nuclear texture feature from the corresponding drop-down list.
   f. Change the ‘Color’ of the symbol filling option to yellow.
   g. Change the color of the nucleus to red. To do this, first check the ‘Color’ check box of the inner sub-ellipse. Next, select the ‘Fixed’ radio/option button to get all nuclei colored in the same color. The color drop-down list will be enabled where you can select the red color.

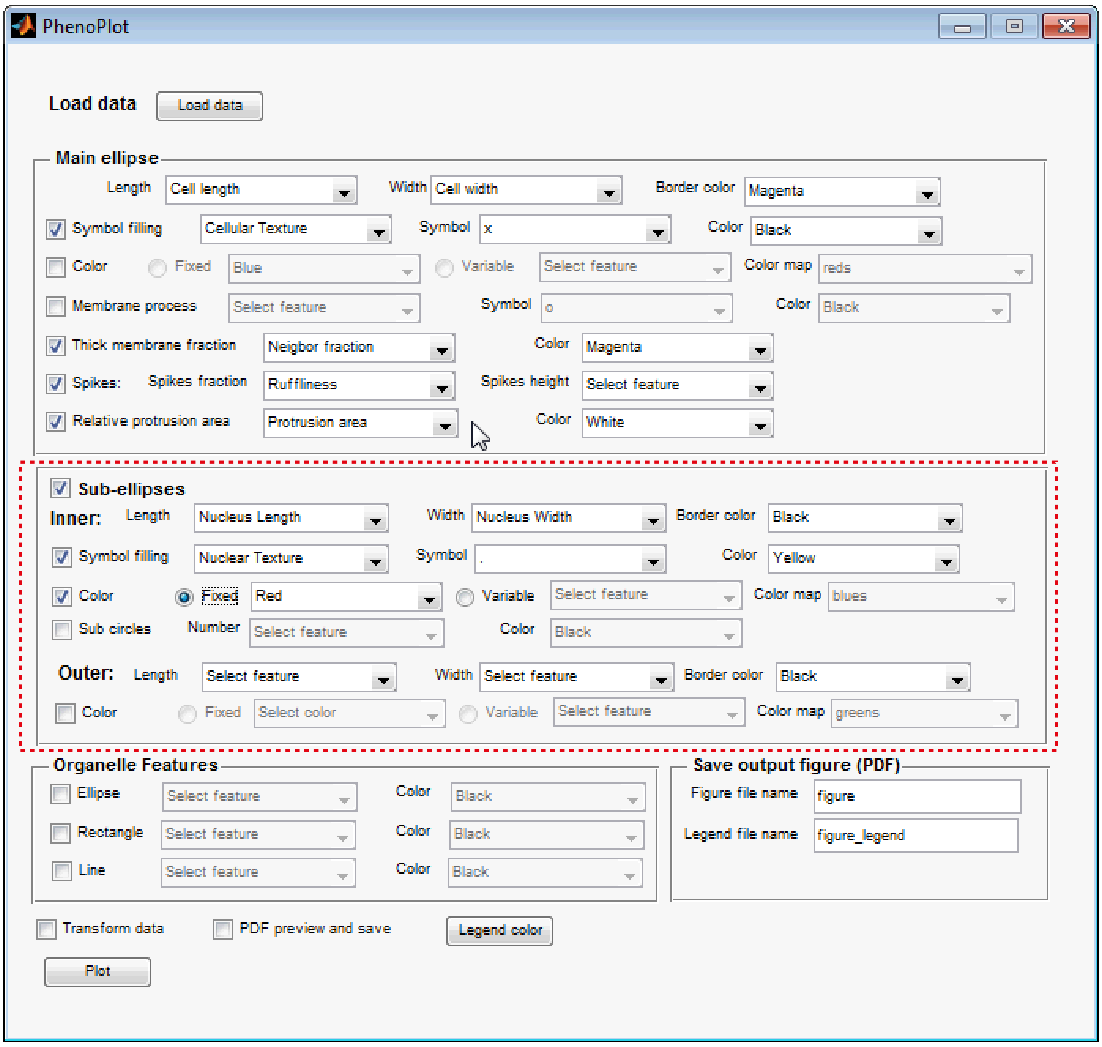

Figure 13: Specifying the Sub-ellipse elements

11- In the ‘Save output figure’ panel type “BCL_clusters” in ‘Figure file name’ text box and type “BCL_clusters_legend” in the ‘Legend file name’ text box (Figure 14). These will be used to save the output figure to the current working folder when ‘PDF preview and save’ option is checked, see next.

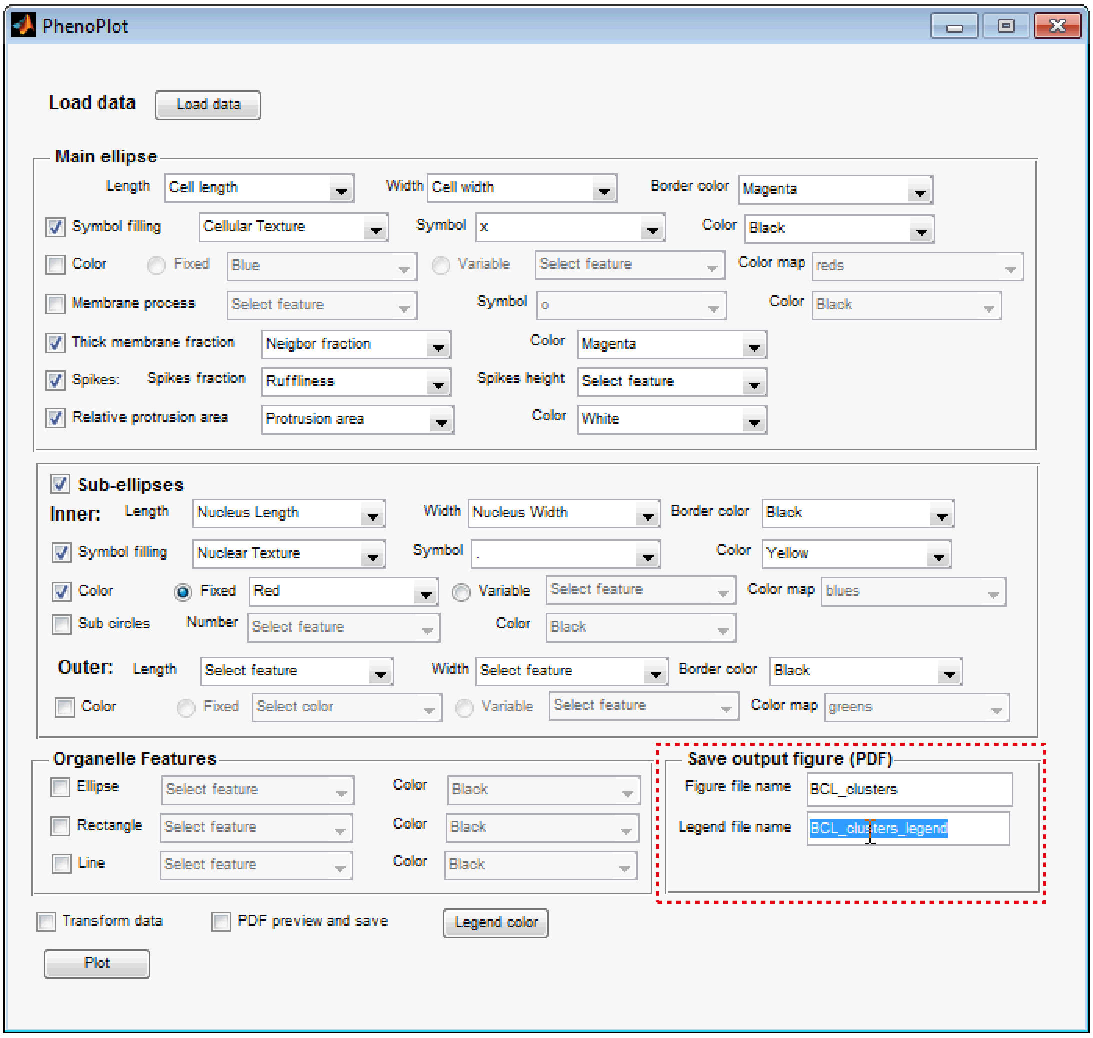

Figure 14: Specifying output figure file names

12- Check ‘PDF preview and save’ check box to save the output figures in PDF format. A preview will open in Acrobat reader when clicking the ‘Plot’ button (Figure 15). Note this will only work if you have Ghostscript installed, if not it is recommended that you save the figures manually in ‘eps’ format.

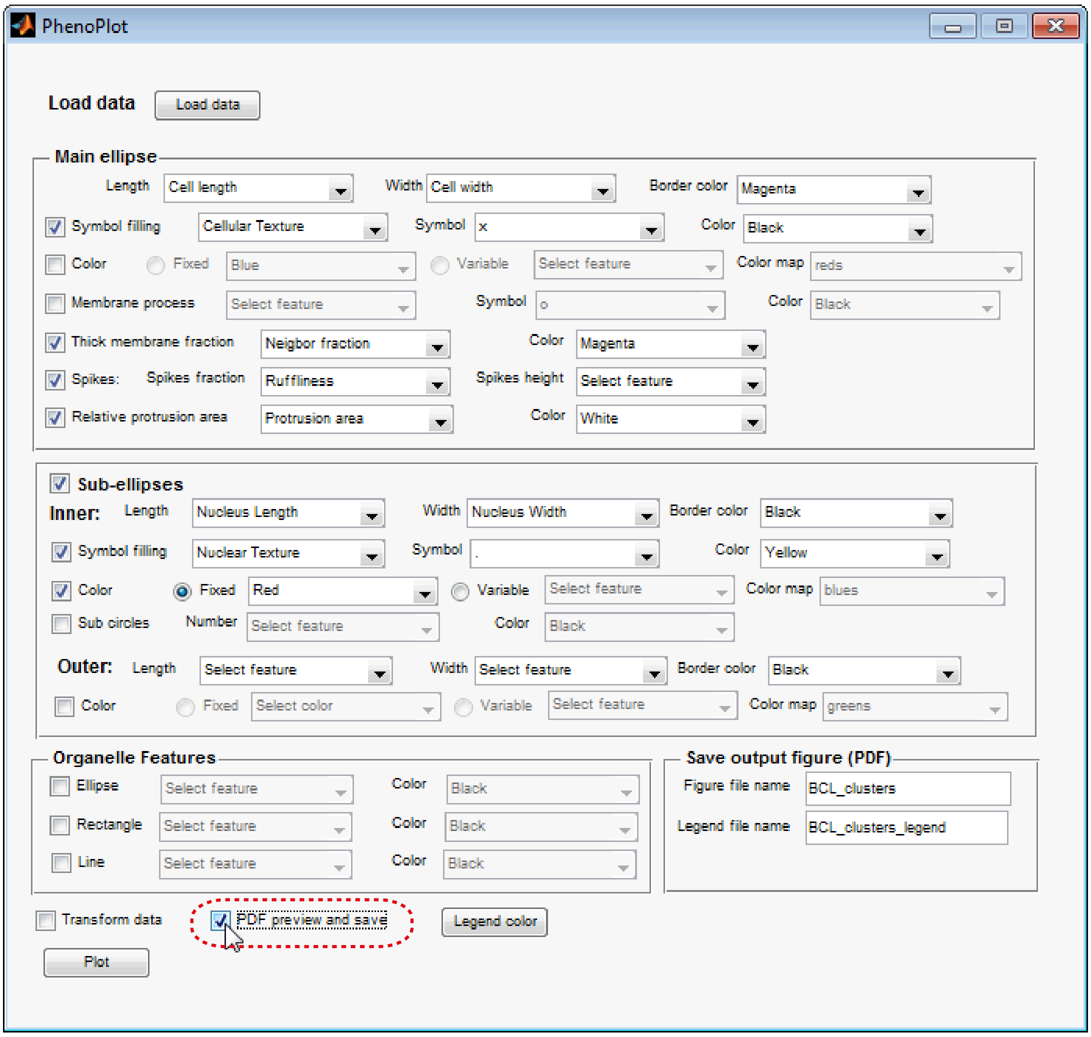

Figure 15: Preview figures in PDF and save them to the current working folder.

13- Finally, we will change the color of the text and the arrows of the legends. Click on the ‘Legend color’ button. This will open a color selection window (Figure 16), select the black color and press ‘OK’ button.

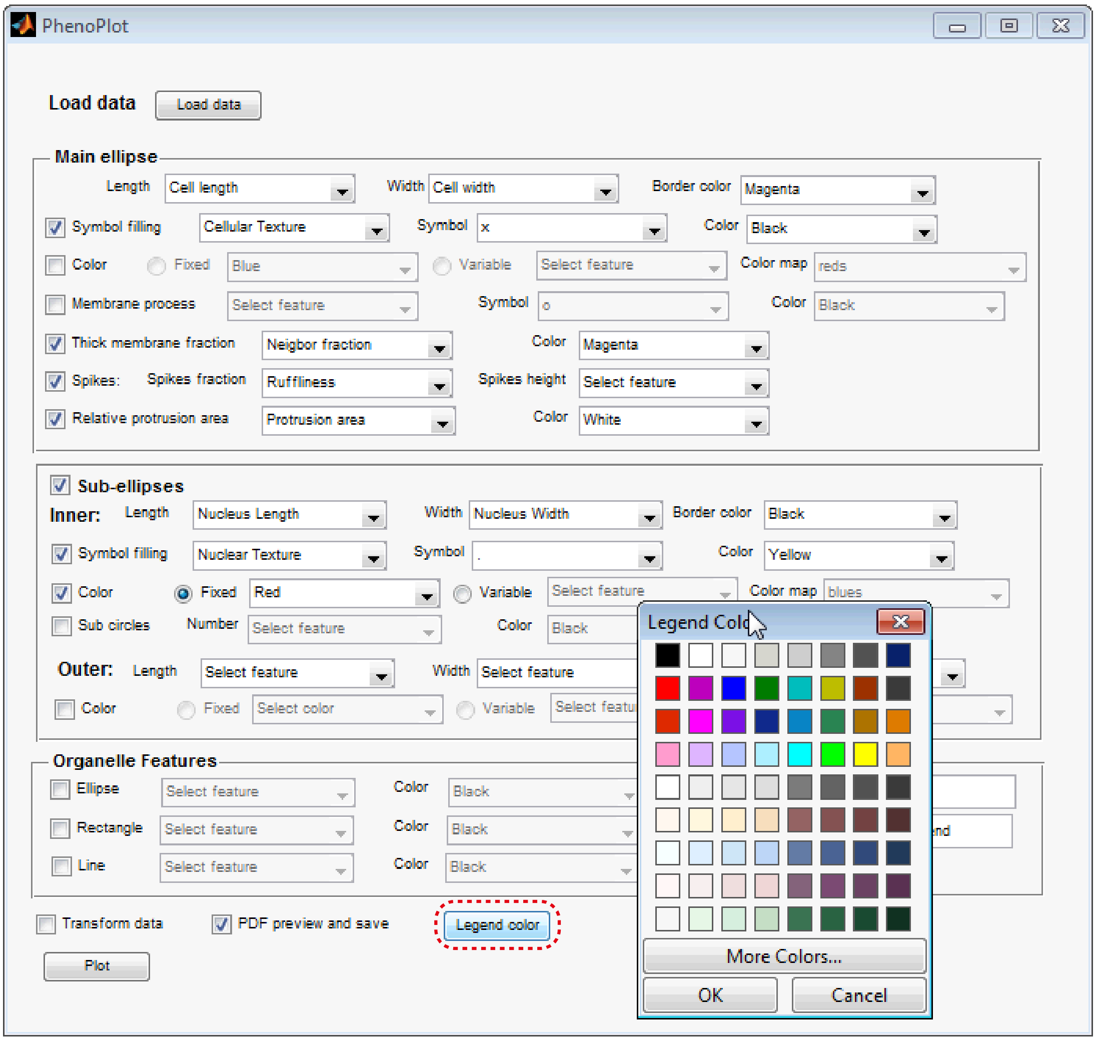

Figure 16: Changing legend color

14- When finished click ‘Plot’ button to generate the figure. The corresponding legend will be plotted automatically in a separate figure where various elements will be labeled using the feature names in BCL_clusters.csv (Figure 17).

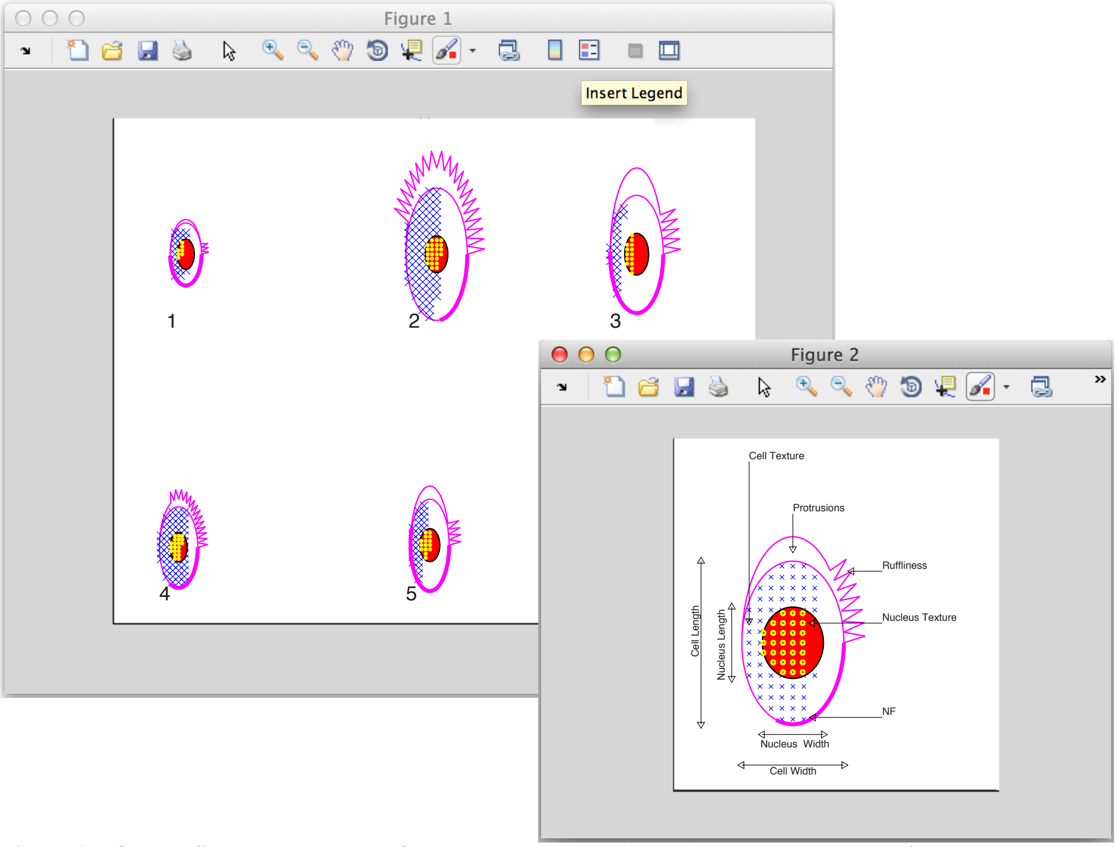

Figure 17: Output figures: PhenoPlots for each data sample (cluster) and the corresponding legend

VIII. Demo 2: Representing various cellular markers

This example will use artificial dataset to illustrate how to use PhenoPlot to represent the average intensity of three fluorescence markers of different cellular regions: cellular marker 1 and 2 and nuclear marker. Here we will assign variable values to the color of main, inner and outer ellipses.

1- Click on the 'Load data' button, then locate demo_markers.csv file from PhenoPlot folder. The feature names in demo_markers.csv will be loaded in the PhenoPlot GUI so that you can select a feature for each element.
2- Specify the dimensions and color for main, inner and outer ellipses as shown in Figure 18.

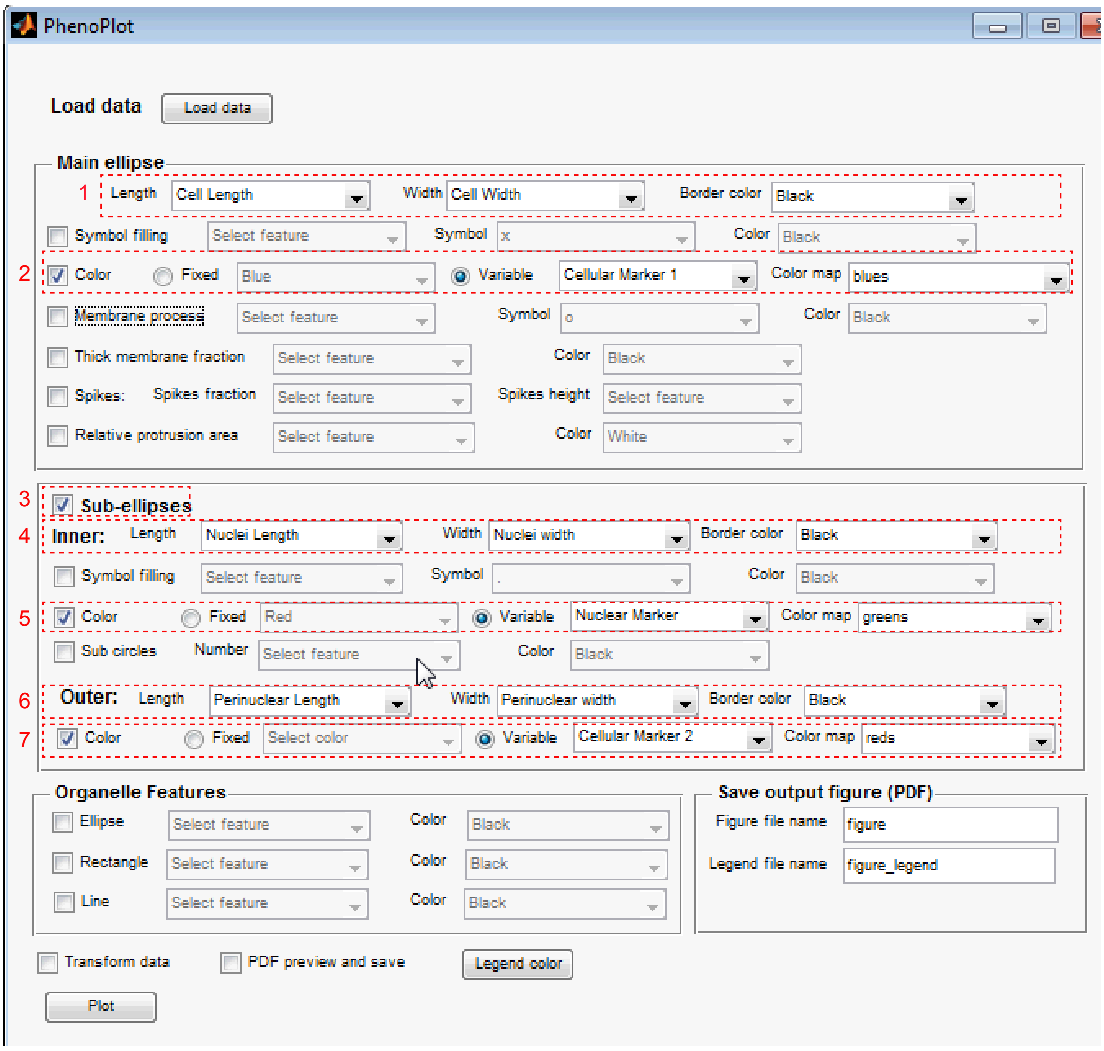

Figure 18: Plotting main and sub-ellipses with variable color.

3- Click the ‘Plot’ button to generate PhenoPlot representation and legend (Figure 19).

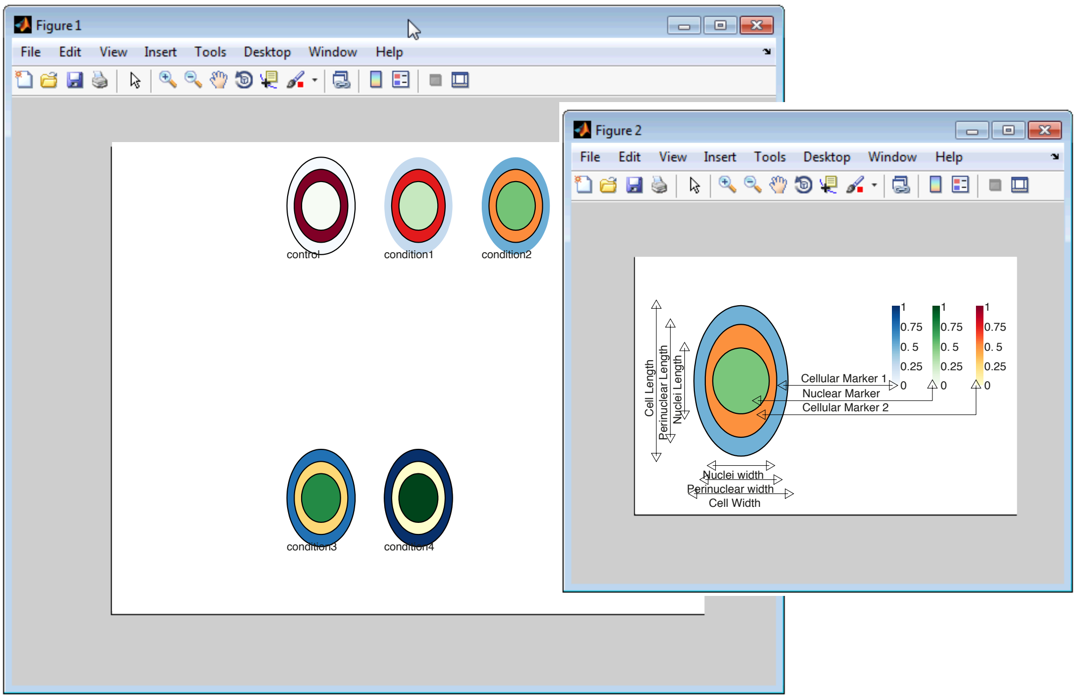

Figure 19: PhenoPlot representation of data in demo_markers.

IX. Using PhenoPlot from the command line

The source code is freely available under the enclosed license. To use PhenoPlot function in your code please refer to PhenoPlot function documentation. You can test the function by running the following command

```
> PhenoPlot ({1:10',1:10'},'Standardize',1)
```

To see example code check the following demo files:
Demo1_Breast_Cancer_Clusters: an example on plotting the average measurements of different clusters using data that describe the shape of different breast cancer cell lines.

Demo2_Breast_Cancer_PCA: plots the average measurements for 18 breast cancer cell lines. The position of each PhenoPlot is based on the first two principal components (where PCA is applied on the same features and standard PCA is normalization applied).

For any enquiry please contact Heba Sailem: heba.sailem @ gmail . com

Heba Sailem, Nov 2014

X. Citing PhenoPlot

When using PhenoPlot, please cite our manuscript
Sailem, Sero, Bakal (2014) Visualizing cellular imaging data using PhenoPlot, Nature Communications

XI. Copyright

Copyright © Heba Sailem and the Institute of Cancer Research, London, UK.
