# Supplementary figures and images for: Visualizing cellular imaging data using PhenoPlot
Source: Nat Commun. 2015 Jan 8;6:5825. doi: 10.1038/ncomms6825 (PMC4354266; doi:10.1038/ncomms6825)

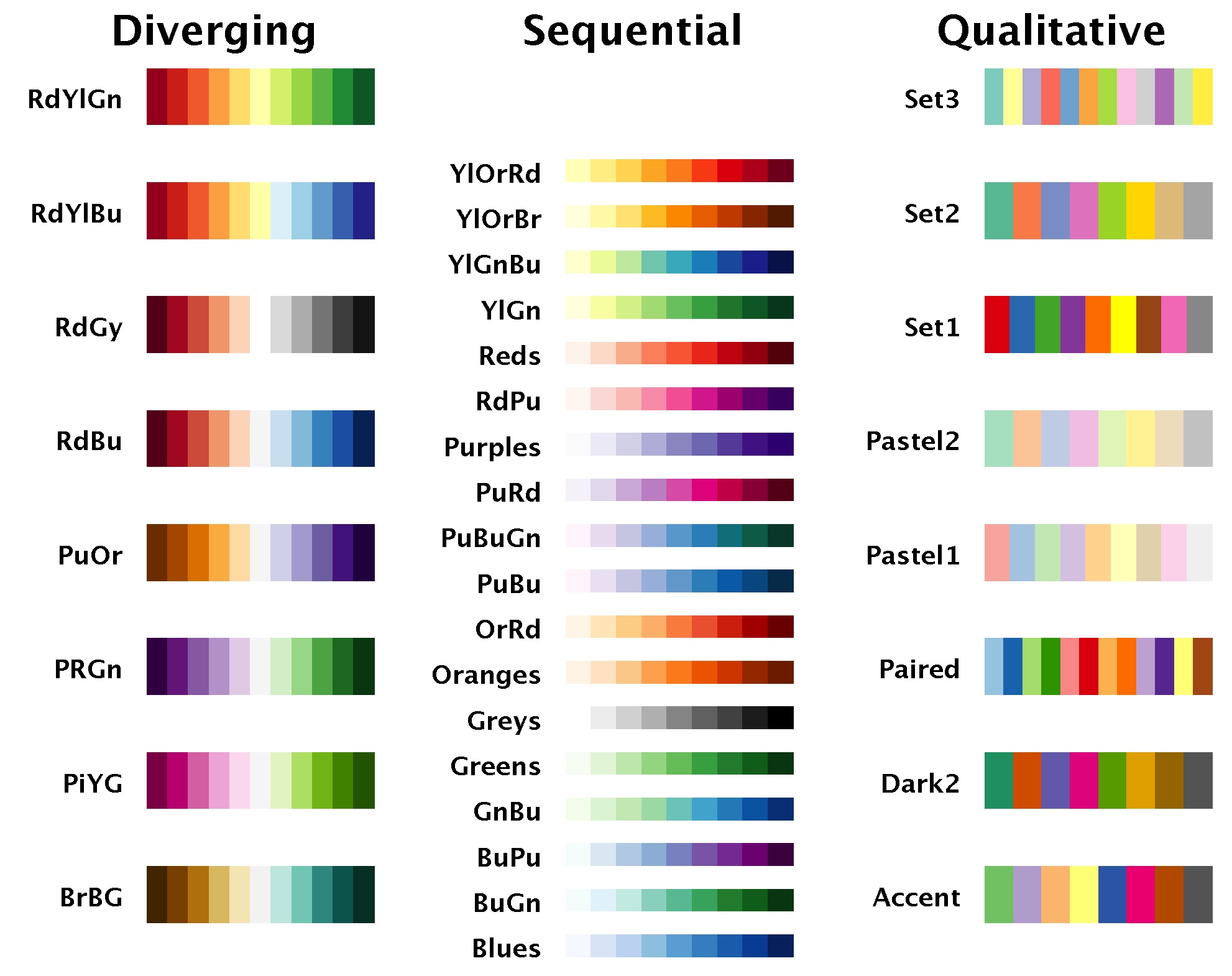

Supplement: Supplementary Software — PhenoPlot is a Matlab toolbox with an interactive Graphical User Interface (GUI) that generates cell-like glyphs from imaging data. The software requires Matlab 2012 and is best-used with data extracted from cellular images, but can be used to represent any numerical data. A guide on using the software both from the command line, and using the GUI, is provided in the file PhenoPlot_manual.pdf. [file ncomms6825-s2.zip › Supplementary software/PhenoPlot/cbrewer/cbrewer_preview.jpg]
